# A Bidirectional Interpolation Method for Post-Processing in Sampling-Based Robot Path Planning

**DOI:** 10.3390/s21217425

**Published:** 2021-11-08

**Authors:** Tae-Won Kang, Jin-Gu Kang, Jin-Woo Jung

**Affiliations:** 1 Department of Artificial Intelligence, Dongguk University, Seoul 04620, Korea; ktw3388@dgu.ac.kr; 2 Department of Computer Science and Engineering, Dongguk University, Seoul 04620, Korea; kanggu12@dongguk.edu

**Keywords:** bidirectional interpolation method, post-processing, RRT-connect, triangular RRT-connect, midpoint interpolation, sampling-based path planning

## Abstract

This paper proposes a post-processing method called bidirectional interpolation method for sampling-based path planning algorithms, such as rapidly-exploring random tree (RRT). The proposed algorithm applies interpolation to the path generated by the sampling-based path planning algorithm. In this study, the proposed algorithm is applied to the path created by RRT-connect and six environmental maps were used for the verification. It was visually and quantitatively confirmed that, in all maps, not only path lengths but also the piecewise linear shape were decreased compared to the path generated by RRT-connect. To check the proposed algorithm’s performance, visibility graph, RRT-connect algorithm, Triangular-RRT-connect algorithm and post triangular processing of midpoint interpolation (PTPMI) were compared in various environmental maps through simulation. Based on these experimental results, the proposed algorithm shows similar planning time but shorter path length than previous RRT-like algorithms as well as RRT-like algorithms with PTPMI having a similar number of samples.

## 1. Introduction

This study deals with the path planning of a mobile robot [1]. Strictly speaking, path planning can be divided into global planning on an entire map and local planning on a portion of the map [2]. In this paper, path planning refers to global planning.

Path planning involves plotting a path that a mobile robot can follow to efficiently move from a starting point to a goal point in Euclidean space, avoiding obstacles, with respect to optimality, clearance and completeness [3]. Optimality refers to always being able to plan a path with the optimal path length. Clearance refers to how low the probability is that the mobile robot will collide with an obstacle. Completeness indicates that a path can always be planned in an environment in which a solution exists.

This paper mainly deals with the sampling-based rapidly-exploring random tree (RRT)-like algorithm [4]. The RRT-like algorithms are being applied in various ways. For example, there is a method that generates an optimal path by applying a triangular inequality [5] and a method applicable to kinodynamic planning [6]. The RRT algorithm can be summarized as a method of planning a path by repeatedly inserting a randomly sampled location as a child node in a tree with the starting point as the root node until reaching the goal point. In this method, the tree trunk extends in the shape of a stochastic fractal and attempts to reach the goal. 

Sampling-based algorithms, including RRT algorithm, have the advantage of being able to plan a path with fewer computations than classical path planning algorithms such as those for visibility-graph-based [7], cell-decomposition-based [8] and potential-field-based [9] methods. However, RRT does not guarantee optimality and probabilistic completeness [4]. Probabilistic completeness means that if the number of sampling nodes is unlimited, completeness is guaranteed, but if the number of sampling nodes is limited, completeness is not guaranteed.

**Figure 1:**
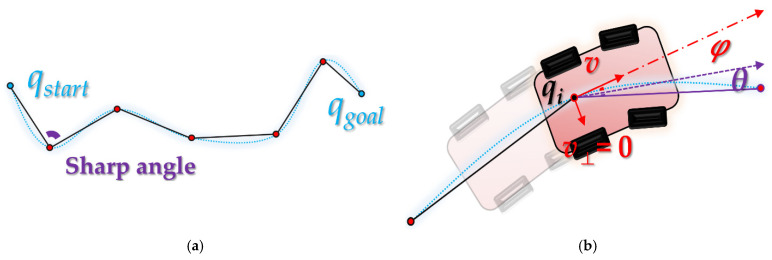
Path of RRT-like algorithms with sharp angle: (**a**) piecewise linear path; (**b**) situation with turning radius *φ* as per the kinematic constraints of mobile robot with velocity *v* at node (turn penalty *θ*).

The purpose of this study is to guarantee completeness, improve optimality and improve collision-avoidance of RRT-like algorithms for path planning. When a path is planned by the RRT algorithm, it has a stochastic fractal shape, and locally it tends to have a piecewise linear shape [10] as shown in Figure 1a. The piecewise linear contour can result in collisions [11] owing to the kinematic constraints [12] of the mobile robot, as shown in Figure 1b.

**Figure 2:**
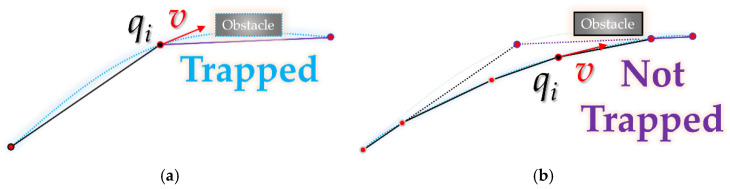
Path needing kinodynamic planning (when a mobile robot with kinematic constraints moves at a velocity *v* at an arbitral point *q_i_*): (**a**) situation in which the robot collides with the obstacle; (**b**) situation in which the obstacle is avoided by increasing way points.

When the mobile robot moves along the planned path or local planning [13] path, it may collide with an obstacle as shown in Figure 2a because of kinematic constraints on the path with sharp angles. As this leads to serious concerns from the perspective of clearance, path planning must also consider kinodynamic planning [6,14,15]. In particular, as there are fewer waypoints on the planned path (a tree node in the RRT algorithm) or, alternatively, the distance between waypoints is high, kinematic error is more likely to occur. This may be exacerbated when the mobile robot travels at high speed and the control error increases.

In this study, given that the RRT algorithm does not guarantee optimality, we aim to generate a path that is closer to the optimum. Simultaneously, the scope of the study includes solving the local clearance problem of the stochastic fractal-shaped path.

However, when the path is first plotted from the starting point to the goal point, only the first complete path is dealt with. That is, the complete path or convergence path that is closer to the optimum through additional sampling after the initial complete path is not considered within the scope of the current study.

The RRT* algorithm, an improved version of the RRT algorithm, further optimizes the convergence path, wherein the convergence rate of the first complete path is higher than that of the RRT algorithm. Consequently, it is possible to plan a path closer to the optimum. However, the amount of computation required to arrive at the convergence path is very high [16]. Recently, the post triangular processing of midpoint interpolation (PTPMI) method has been proposed to solve this problem [17]. Therefore, in this study, we prioritize finding a solution, which is the advantage of the sampling-based algorithm. In addition, the purpose of this study is to generate a path that is closer to the optimum for a similar computation time without significantly increasing the amount of computation compared to related studies.

We propose the bidirectional interpolation method for post-processing, which allows the RRT algorithm to plan a path that is closer to optimal and to mitigate the local clearance problem.

Visibility-graph-based path planning creates an optimal path. However, in order to make this path clearer in a real environment, it is necessary to use a sensor device with high resolution. The proposed method in this paper can generate a path that is close to optimal even in sensor equipment with low resolution. Therefore, the cost for the sensor could be saved.

The overall structure of this paper is as follows. Section 2 deals with related works. Section 2.1 deals with classical path planning and Section 2.2 deals with the sampling-based path planning algorithm. Section 3 deals with the proposed method (bidirectional interpolation method for post-processing). Section 4 deals with experimental results in which the path lengths and planning times are compared and analyzed through simulation to verify the performance of the proposed method. 

## 2. Related Works

### 2.1. Classical Path Planning

#### 2.1.1. Visibility Graph

The visibility graph algorithm guarantees optimality and completeness, but the clearance is very low. The cell decomposition algorithm guarantees completeness and high clearance, but does not guarantee optimality. The potential field algorithm has very high clearance, but cannot guarantee completeness and optimality by local minima [9].

In particular, as the visibility graph algorithm guarantees optimality in 2D configuration space [18], it is often used as a comparative experiment target for path planning method studies. We also compare the RRT algorithm post-processed using the proposed method to the visibility graph in terms of path length, in order to gauge their relative optimality.

**Figure 3:**
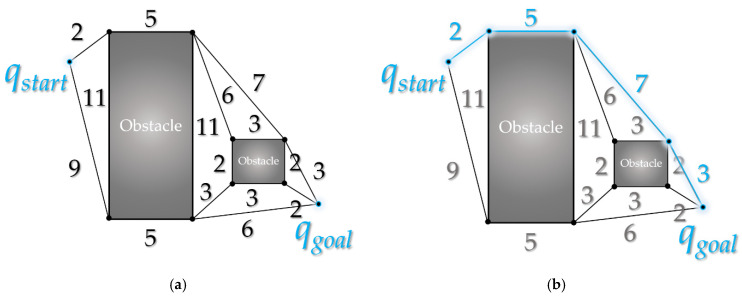
Overview of visibility graph: (**a**) graph (the number on the edge is the length of that edge) connecting start point *q_start_*, goal point *q_goal_* and the vertices of all obstacle polygons; (**b**) shortest path from start point *q_start_* node to goal point *q_goal_* node.

The visibility graph algorithm was proposed by Tomas Lozano-Perez in 1979. As shown in Figure 3a, the start point, goal point and the vertices of all obstacle polygons are connected in a graph form. As shown in Figure 3b, it guarantees optimality by searching for the shortest path from the node at the starting point to the node at the goal. At this time, the case with an edge connecting a node and a node passing through an obstacle is excluded.

#### 2.1.2. Limitation of Classical Path Planning

All the classical path planning methods, including the visibility graph algorithm, have their own advantages and disadvantages, but a common limitation is that they are difficult to apply to a dynamic environment because they involve a large amount of computation.

The path planning algorithms of the classical method introduced above plan the path using obstacle area information, unlike the sampling-based algorithm. As the visibility graph algorithm and the cell decomposition algorithm plan a path using the vertices of the obstacle polygon, the amount of computation becomes very large when the number of vertices of the entire obstacle is large. In addition, given that the visibility graph algorithm and cell decomposition algorithm can be applied only when the shape of the obstacle is polygonal, polygon approximation is required when a curved obstacle is given as an input value in a vector map rather than a grid map [19,20].

The precision of modern mobile robot sensing technology and equipment (such as radar and LiDAR) is very high, so obstacles can be mapped with high resolution (the number of polygonal vertices of obstacles is proportional to the sensing precision) [21]. Therefore, path planning through classical methods such as visibility graph algorithm is difficult to apply to dynamic environments. One solution is to reduce the computational amount of the classical method by reducing the number of obstacle polygon vertices through the polygon approximation algorithm. However, this distorts the actual shape, and therefore unintentional merging between polygons or obstacle collision problems caused by distorted areas can cause fatal problems in route planning.

Unlike the classical method, the sampling-based method does not actively use the obstacle area information for path planning; it is only used for obstacle collision inspection. Therefore, the amount of computation does not increase significantly depending on the type of obstacle. In other words, it is suitable as a modern mobile robot route planning method because it can plan a route in a short time even in a dynamic environment mapped at a high resolution.

### 2.2. Sampling-Based Path Planning Algorithms

Sampling-based path planning methods include the RRT algorithm and the probabilistic roadmap method (PRM) algorithm [22]. Well-known improvements to the RRT algorithm include RRT-connect [23], RRT* [14], informed RRT [24] and quick*-RRT [10] algorithms. This section deals only with RRT and RRT-connect algorithms.

As the purpose of this paper is specifically to improve the performance of the first complete path of the RRT algorithm, research on the efficient convergence path or the RRT* algorithm for improving convergence speed or the informed RRT algorithm for improving random sampling is not covered in this paper.

#### 2.2.1. Rapidly Exploring Random Tree (RRT)

The RRT algorithm is a representative algorithm of the sampling-based path planning method, and was proposed by Steven M. LaValle in 1998 [4]. It is useful for path planning, considering the conditions of non-holonomic constraints, and is designed to have high degrees of freedom [6].

**Figure 4:**
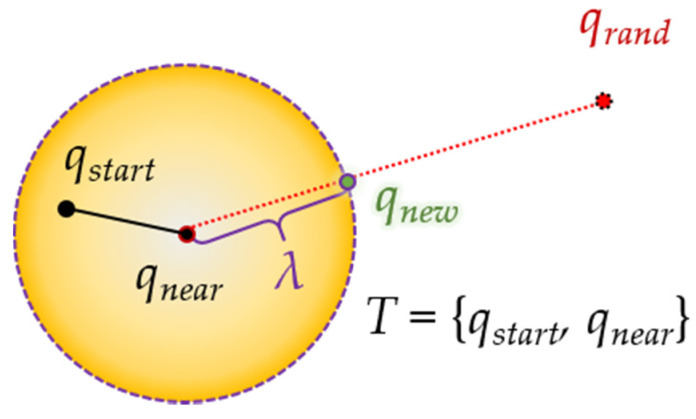
Process of RRT algorithm. A new node is created at location *q_new_* separated by step length *λ* in the direction of the random sampling position *q_rand_* based on the random sampling position *q_rand_* and the nearest node (position) *q_near_* in tree *T* with start point *q_start_* as the root node.

When a random sample is generated in the configuration space, as shown in Figure 4, the node closest to the location of the random sample is found among the nodes constituting the tree with the starting point as the root node. A new node is created at a position away from the node by step length (*λ*) in the direction of the random sample position and inserted into the tree. If the random sample position is closer than step length, a new node is created at the random sample position and inserted into the tree. This tree expansion process is repeated until the destination is reached.

#### 2.2.2. RRT-Connect

As samples appear with the same probability in all regions, path planning through the RRT algorithm may have the disadvantage that the tree easily extends in several random directions irrespective of the destination, resulting in a longer convergence time and inefficiency. The RRT-connect algorithm [23] proposed by James J. Kuffner Jr. in 2000 aims to compensate for this disadvantage by adopting two major new strategies.

The first idea is swapping. It involves designating a starting point and a target point as each root node and alternately extending in the direction of each other. This prevents the tree from extending in a direction independent of its destination and reduces the time required to search for a path.

**Figure 5:**
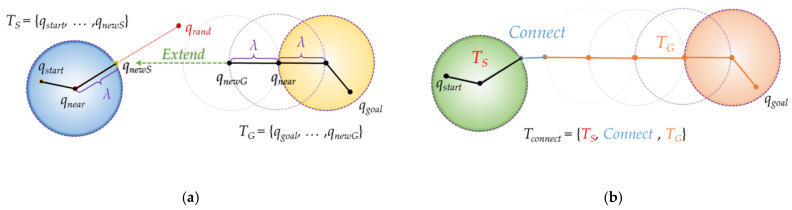
Process of RRT-connect algorithm: (**a**) extend from tree *T_b_* with root as goal position *q_goal_* to tree *T_a_* with root as start position *q_start_* (*T_a_*’s *q_near_* extends in *T_a_*’s *q_newA_* direction); (**b**) as the paths *P_a_* and *P_b_* created in each tree are connected (“Connect”) to each other, path *P_merged_* is created.

The second idea is extending. This means that when the tree is extended, it continues to extend to the tree on the other side if no collision with an obstacle occurs. As shown in Figure 5, if there is no collision with an obstacle when a sample is generated, the tree continues to expand as much as the step length in the direction of the opposite tree, so that the destination can be reached faster.

Through this idea, the path planning through the RRT-connect algorithm can find the first complete path at a much higher speed than the existing RRT algorithm.

#### 2.2.3. Triangular-RRT-Connect 

**Figure 6:**
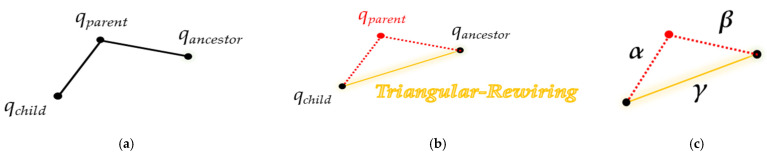
Overview of “Triangular-Rewiring” method: (**a**) example tree, (**b**) result of “Triangular-Rewiring”, (**c**) applied trigonometric inequality (*α* + *β* > *γ*).

Triangular-RRT-Connect [25] is a method that applies the triangular-rewiring method in the extend and connect stages of RRT-connect. Triangular-rewiring uses the principle of triangular inequality. As shown in Figure 6, when there are *q_child_*, *q_parent_* which is the parent node of *q_child_* and *q_ancestor_* which is the parent node of *q_parent_*, triangular-rewiring eliminates the path through the *q_parent_* node when there is no obstacle between *q_child_* and *q_ancestor_* and connects directly between *q_child_* and *q_ancestor_*.

**Figure 7:**
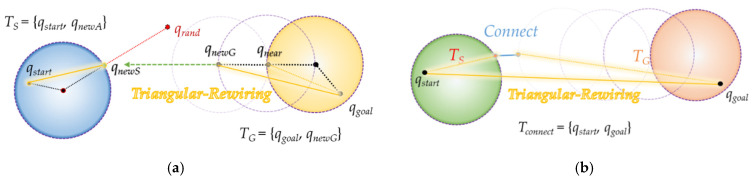
Extend and connect in triangular-RRT-connect: (**a**) extend method in triangular-RRT-connect; (**b**) connect method in triangular-RRT-connect.

Figure 7 shows “extend” and “connect” of triangular-RRT-connect. In triangular-RRT-connect, there is a tree extending from the goal point and a tree extending from the start point. In Figure 7a, *T_S_* and *T_G_* are trees extending from the start point and the goal point, respectively. Extend occurs at *T_G_* when sampling is performed at a random location *q_rand_* and a new node, *q_newS_*, is created based on *T_S_*. At this time, the triangular-rewiring method works on all nodes generated during the “extend” process. Figure 7b is connect in triangular-RRT-connect. After the two trees are connected, the triangular-rewiring method is applied to the merged tree. *T_connect_* is a tree where *T_S_* and *T_G_* are connected.

## 3. Bidirectional Interpolation Method for Post-Processing

### 3.1. Forward Interpolation Process

**Figure 8:**
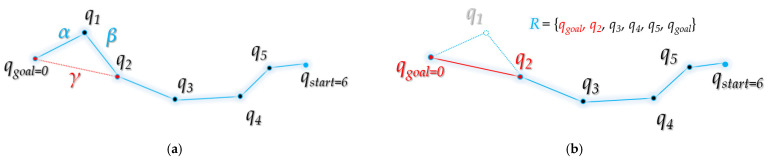
Overview of rewiring step in forward interpolation process: (**a**) line *γ* from node *q*_0_ to its grandparent node *q*_2_ in tree *R* is collision-free (distance: *γ* < *α* + *β*); (**b**) rewiring: grandparent node *q*_2_ of node *q*_0_ is connected to *q*_0_ as parent node, and origin parent node *q*_1_ is excluded from tree *R*.

As shown in Figure 8a, when there is no obstacle between the newly inserted node and its grandparent node (collision-free), as shown in Figure 8b, a connection is made between the newly inserted node and its grandparent node, while its parent node is excluded (rewiring). Based on the trigonometric inequality property, this can be corrected to a path that is closer to the optimum than in the existing RRT algorithm.

**Figure 9:**
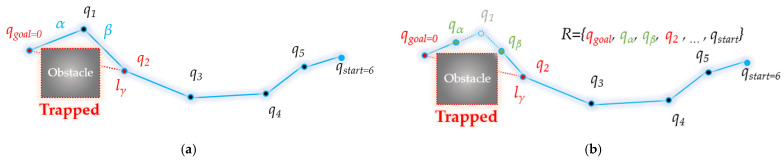
Overview of interpolation step in forward interpolation process: (**a**) line *γ* from node *q*_0_ to its grandparent node *q*_2_ in tree *R* is trapped; (**b**) interpolation: node *q_a_* between node *q*_0_ and node *q*_1_ and node *q_b_* between node *q*_1_ and node *q*_2_ are created. After connecting the parent node of *q*_0_ with *q_a_*, the parent of *q_a_* with *q_b_* and the parent of *q_b_* with *q*_2_, node *q*_1_ is excluded.

If the current path is not collision-free from obstacle, as shown in Figure 9, the piecewise linear local path created between the node, its parent node and its grandparent node is optimized through interpolation. In this process, new nodes are interpolated into the existing path to deviate from the piecewise linear path, making it possible to create a smooth path.

Quick-RRT* [10] and triangular-RRT-connect [25] algorithms aim to create a path that is close to optimal using triangular inequality. However, as the node is deleted in the process, the distance between the waypoints on the planned path is longer than that of the RRT algorithm, so the sharp angle on the path line is deepened.

Forward interpolation process is effective for all path planning algorithms, such as RRT algorithm, where optimality is not guaranteed and a local piecewise linear path appears. After the route is planned, it can be applied as a post-processing technique.

Compared to the classical path planning methods [3], the major advantage of the sampling-based path planning method is the high planning speed owing to a small amount of computation, so there is a prerequisite that the amount of added computation should not be large compared to that required by the existing RRT algorithm.

**Figure 10:**
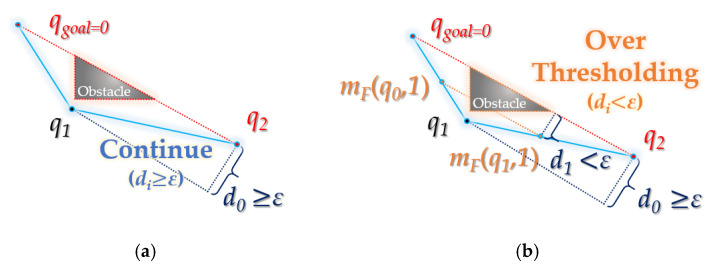
Condition of interpolation step at forward interpolation process: (**a**) interpolation continues**:** the height *d*_0_ of a triangle made from waypoint *q*_0_, *q*_1_ and *q*_2_ in random path is higher than *ε*; (**b**) interpolation break**:** the height *d*_1_ of a triangle made from midpoint *m_F_*(*q*_0_,1) (between *q*_0_ and *q*_1_)*_,_* node *q*_1_ and midpoint *m_F_*(*q*_1_,1) (between *q*_1_ and *q*_2_) is less than *ε*.

Forward interpolation process was designed based on the polygon approximation algorithm [18,26]. As shown in Figure 10, the constant value (*ε* > 0) of *ε* (the threshold of minimum collision stability) determines how closely the path is approximated to the obstacle.

*d_n_* in Figure 10 follows Equation (1):

(1) $$d_{n}\left(q_{i}\right)=\{\begin{array}{c} \;\;\;\;\;\;\;\;\;\;\;\;\;\;\;\left(d_{n-1}\left(q_{i}\right)\right)/2,\;\;\;\;n>0\\ \;\;\left(2\sqrt{s\left(s-\alpha \right)\left(s-\beta \right)\left(s-\gamma \right)}\right)/\gamma ,\;\;n=0 \end{array}\;\left(s=\left(\alpha +\beta +\gamma \right)/2\right)$$

For an arbitrary waypoint *q_i_*, the value of *d* decreases by 1/2 as interpolation proceeds (*n*). The initial value *d*_0_ is the height of the triangle consisting of the length *α* from *q_i_* to the next waypoint of *q_i_*, the length *β* from the next waypoint of *q_i_* to the next waypoint of the next waypoint of *q_i_* and the length *γ* from *q_i_* to the next waypoint of the next waypoint of *q_i_*(*γ < α + β*). The value of *d_n_* becomes (*d_n_* − 1)/2. As the path gets closer to the obstacle as *d* gets smaller, it is compared to *ε* functions as a measure to confirm clearance.

**Figure 11:**
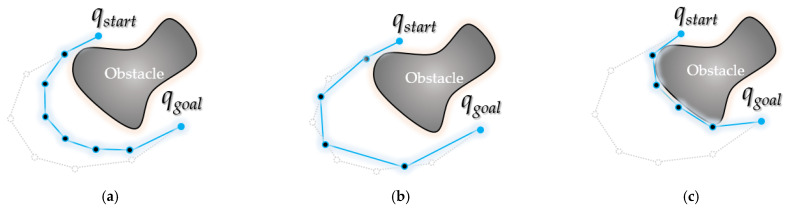
Difference according to *ε* value in forward interpolation process: (**a**) result when the value of *ε* is equal to random value *n*; (**b**) result when *ε* value is more than random value *n*; (**c**) result when *ε* value is less than random value *n*.

However, optimality and clearance are conflicting properties; as shown in Figure 11, the smaller the *ε* value (the minimum value of *ε*: 0), the higher the optimality and the lower the clearance. Conversely, the larger the *ε* value, the higher the clearance and the lower the optimality. Therefore, *ε* should be set to an appropriate value based on the environment.

#### 3.1.1. Pseudocode of Forward Interpolation Process

Forward interpolation process is a post-processing method applied after the path is planned in RRT-like algorithms. Mathematical modeling is based on a two-dimensional Euclidean space.

Algorithm 1 presents the pseudocode of forward interpolation process. Two functions can be called internally: post triangular (Algorithm 2) and interpolation (Algorithm 3).
**Algorithm 1** Pseudocode of Forward Interpolation Process.**Input:***R* ← path from {*RRT/RRT-Connect/Tri-RRT/Tri-RRT-Connect*/…}*C* ← position set of all (measured) boundary points in all (known) obstacles*ε* ← threshold value of minimum clearance**Output:***R* ← modified path *R***Initialize:***f_modify_* ← ***true*****Procedure***ForwardInterpolationProcess***Begin**1**While***f_modify_***Do**2    *f_modify_* ← ***false*** //**is the path modified**3    *t* ← 0  //**index of the currently focused point**4    *q_child_* ← first point in *R*5    *q_parent_* ← next point of *q_child_* in *R*6    **While not** [*q_parent_* is last point in *R*] **Do**7       *q_ancestor_* ← next point of *q_parent_* in *R*8       **If not**
*isTrapped*(*q_child_*, *q_ancestor_*, *C*) **Then**  *R* ← *postTriangular*(*R*, *ε*, *t*, *f_modify_*)9       **Else**10          *R ← interpolation*(*R*, *C*, *ε*, *t*, *f_modify_*)11          *q_child_* ← *t*-th point in *R*12       *q_parent_* ← next point of *q_child_* in *R***End**
**Algorithm 2** Pseudocode of the Post Triangular Function.**Input:***R* ← path *R* from *postTriProcOfMidInterpolation**t* ← point index *t* from *postTriProcOfMidInterpolation**f_modify_* ← boolean *f_modify_* from *postTriProcOfMidInterpolation***Output:***R* ← modified path *R**f_modify_* ← result of boolean *f_modify_*  //**return by reference****Procedure***postTriangular***from***ForwardInterpolationProcess***Begin**1*q_child_* ← *t*-th point in *R*2*q_parent_* ← next point of *q_child_* in *R*3*q_ancestor_* ← next point of *q_parent_* in *R*4*R* ← **Delete** path<*q_child_*, *q_parent_*> and path<*q_parent_*, *q_ancestor_*> from *R*5*R* ← **Insert** path<*q_child_*, *q_ancestor_*> to *R*6*f_modify_* ← ***true*****End**

**Algorithm 3** Pseudocode of the Interpolation Function.
**Input:**
*R* ← path *R* from *ForwardInterpolationProcess**C* ← position set *C* from *ForwardInterpolationProcess**ε* ← threshold value *ε* from *ForwardInterpolationProcess**t* ← point index *t* from *ForwardInterpolationProcess**f_modify_* ← boolean *f_modify_* from *ForwardInterpolationProcess*
**Output:**
*R* ← modified path *R**t* ← updated point Index *t*  //**return by reference***f_modify_* ← result of boolean *f_modify_*  //**return by reference**
**Initialize:**
*q_child_* ← *t*-th point in *R**q_parent_* ← next point of *q_child_* in *R**q_ancestor_* ← next point of *q_parent_* in *R*

**Procedure**
*interpolation*
**from**
*ForwardInterpolationProcess*

**Begin**
1*d* ← altitude of triangle consisting of *q_child_*, *q_parent_*, and *q_ancestor_* with base<*q_child_*, *q_ancestor_*>2*m_a_* ← midpoint between *q_child_* and *q_parent_*3*m_b_* ← midpoint between *q_parent_* and *q_ancestor_*4
**While**
***true***
**Do**
5    **If** *d* **>=** *ε* **Then**6       **If not** *isTrapped*(*m_a_*, *m_b_*, *C*) **Then**7          *R* ← **Delete** path<*q_child_*, *q_parent_*> and path<*q_parent_*, *q_ancestor_*> from *R*8          *R* ← **Insert** path<*q_child_*, *m_a_*>, path<*m_a_*, *m_b_*>, and path<*m_b_*, *q_ancestor_*> to *R*9          *f_modify_* ← ***true***10          ***Break***11       **Else**12          *d* ← *d*/213          *m_a_* ← midpoint between *m_a_* and *q_parent_*14          *m_b_* ← midpoint between *m_b_* and *q_parent_*15    **Else**16       *t* ← *t* + 117       ***Break***
**End**


The input value of forward interpolation process consists of the path *R* planned through the RRT-like algorithms, the obstacle area information *C* and the threshold value *ε* of the minimum clearance.

*f_modify_* is a variable that checks whether the input path *R* has been modified by this method, and if the path is modified even once, the entire process is repeated. If path correction does not occur when the process is repeated, the algorithm is terminated. *t* refers to the index of the waypoint of *R* that is currently focused. That is, if *t* is 0, it refers to the starting point, which is the first point of *R*.

In *R*, when the first starting point is *q_child_*, the next point is *q_parent_* and the next point after that point is called *q_ancestor_*, the algorithm checks whether the line between *q_child_* and *q_ancestor_* is collision-free (*isTrapped*() function). If it is collision-free, the *postTriangular*() function is called; if not, the *interpolation*() function is called. The *postTriangular*() function connects *q_child_* and *q_ancestor_*, and *q_parent_* is excluded from the existing path. The *interpolation*() function finds a random point between (*q_child_* and *q_parent_*) and (*q_parent_* and *q_ancestor_*) that is collision-free when connected (interpolation), and rewires *q_child_*, *q_ancestor_* and the two points found. If *R* and *t* are updated by the *postTriangular*() or *interpolation*() function, *q_child_* (the t-th waypoint of R), *q_parent_* and *q_ancestor_* are updated accordingly. If *q_parent_* is the last point in *R*, *f_modify_* is checked. Otherwise, the above process is repeated again for the updated *q_child_* and *q_ancestor_*.

Here, the path modification by the *postTriangular*() function deletes the existing waypoints and creates a path that is close to optimal, but has the effect of sharpening the path shape. Path modification by the *interpolation*() function has the effect of creating a path that is close to an optimal path and smoothing the path shape while adding/inserting new waypoints between existing waypoints. For creating a path that is close to optimal, the *postTriangular*() function modifies the path more efficiently than the *interpolation*() function.

#### 3.1.2. Pseudocode of the Post Triangular Function from Forward Interpolation Process

The input value of the *postTriangular*() function consists of the path *R*, the waypoint index *t* and *f_modify_*, which states whether the path has been modified by forward interpolation process.

Rewiring is performed on the *t*-th waypoint *q_child_* of *R*, the next point *q_parent_* and the next point *q_ancestor_* of *q_parent_*. First, the path between *q_child_* and *q_parent_* and the existing path between *q_parent_* and *q_ancestor_* are deleted. Then, the path between *q_child_* and *q_ancestor_* is inserted. Finally, *f_modify_* returns ‘*true*’ because the path has been modified.

#### 3.1.3. Pseudocode of Interpolation Function from Forward Interpolation Process

**Figure 12:**
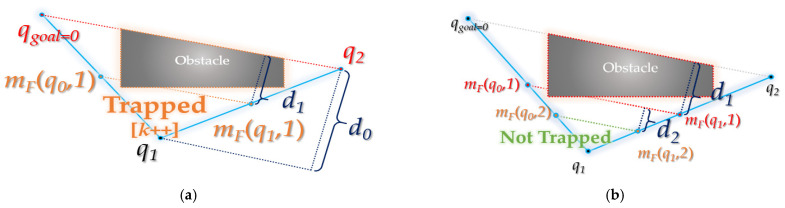
Details of forward interpolation process: (**a**) when the midpoint *m_F_*(*q*_0_*,*1) of the waypoints *q*_0_, *q*_1_ and the midpoint *m_F_*(*q*_1_,1) of *q*_1_, *q*_2_ are not collision-free from the obstacle; (**b**) when the midpoint *m_F_*(*q*_0_,2) of the midpoint *m_F_*(*q*_0_,1), *q*_1_ and the midpoint *m_F_*(*q*_1_,2) of midpoint *m_F_*(*q*_1_,1), *q*1 are not collision-free from the obstacle.

As shown in Figure 12, the interpolation of forward interpolation process is performed at three points (random interpolation point (*q*_0_), the next interpolation point (*q*_1_) and the next interpolation point (*q*_2_) of point *q*_1_). It aims to find the interpolation point (*m_F_(q*_0_*), m_F_(q*_1_)) that is collision-free from the obstacle between the waypoints (*q*_0_*~q*_1_, *q*_1_*~q*_2_) while descending in the direction of the midpoint (*q*_1_).

The interpolation point *m_F_* follows Equations (2) and (3):

(2) $$\xi^{n}\left(q_{i}\right)∶=\{\begin{array}{r} \overset{n}{\left(\overbrace{\xi \circ \xi \circ \ldots \circ \xi }\right)}\left(q_{i}\right),\;n>0\\ q_{i},\;n=0 \end{array}$$

First, *ξ*() is a function that receives a random node as a variable and returns the parent node of that node. The *n*-th square of *ξ*() (*n* ≥ 0) can be expressed as in Equation (2), when *n* = 0, $\xi^{0}\left(q_{i}\right)∶=q_{i}$ holds.

(3) $$m_{F}\left(q_{i},k\right)=\{\begin{array}{c} \left(\frac{m_{F}\left(q_{i},k-1\right)\cdot x+\zeta \left(q_{i}\right)\cdot x}{2},\frac{m_{F}\left(q_{i},k-1\right)\cdot y+\zeta \left(q_{i}\right)\cdot y}{2}\right),\;\;k>0\\ \;\;\;\;\;\;\;\;\;\;\;\;\;\;\;\;\;\;\;\;\;\;\;\;\;\;\;\;\;\;q_{i},\;\;k=0 \end{array}\left(k\in \mathbb{N}\right)$$

When the *k*-th interpolation point of a random waypoint *q_i_* is *m_F_*(*q_i_*,*k*), the 0-th interpolation point itself becomes *q_i_*. The first interpolation point is the midpoint of *q_i_* and the next point *ξ*(*q_i_*) of *q_i_*, and the second interpolation point becomes the midpoint of *m_F_*(*q_i_*,1) and *ξ*(*q_i_*). That is, *m_F_*(*q_i_*, *k*) (*k* > 0) becomes the midpoint between *m_F_*(*q_i_*, *k* − 1) and *ξ*(*q_i_*). At this time, *d* becomes (*d_k_*_−1_)/2.

Algorithm 3 is the pseudocode of the *interpolation*() function in forward interpolation process.

The input value of the *interpolation*() function of forward interpolation process consists of the path *R*, the obstacle area information *C*, the waypoint index *t* and whether to modify the path *f_modify_* from in forward interpolation process. 

A triangle is made of three waypoints (the *t*-th waypoint *q_child_* of *R*, the next point *q_parent_* of *q_child_* and the next point *q_ancestor_* of *q_parent_*), and the height *d* of the triangle can be found. *m_a_* is the midpoint of *q_child_* and *q_parent_*, and *m_b_* is the midpoint of *q_parent_* and *q_ancestor_*. If the path between *m_a_* and *m_b_* is collision-free from the obstacle (*isTrapped*()), the existing path between *q_child_* and *q_parent_* is deleted and the path between *q_child_* and *m_a_*, the path between *m_a_* and *m_b_* and the path between *m_b_* and *q_ancestor_* are inserted. Furthermore, as the path has been modified, *f_modify_* becomes ‘*true*’, returns it, and the function ends. If the distance between *m_a_* and *m_b_* is not collision-free from obstacles, the value of *d* is 1/2, *m_a_* is the midpoint of *m_a_* and *q_parent_*, and *m_b_* is updated to the midpoint of *m_b_* and *q_parent_*, so it must be determined whether *m_a_* and *m_b_* are collision-free from obstacles.

This iterative process proceeds until a case is found in which *m_a_* and *m_b_* are collision-free from obstacles or *d* becomes smaller than *ε*. If *d* becomes smaller than *ε*, the value of *t* is increased by 1 and the function is terminated.

### 3.2. Backward Interpolation Process

**Figure 13:**
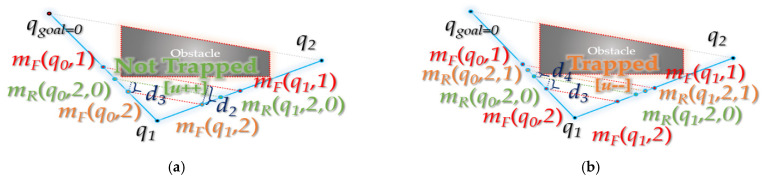

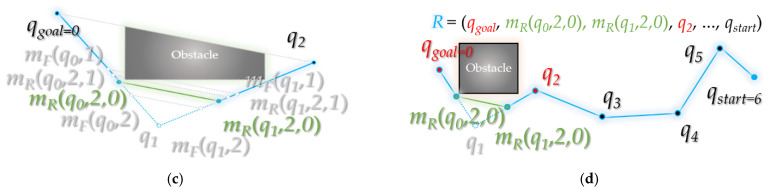
Details of backward interpolation process: (**a**) the path between midpoint *m_R_*(*q*_0_,2,0) of interpolation point *m_F_*(*q*_0_,1), *m_F_*(*q*_0_,2) for existing *q*_0_~*q*_1_ and midpoint *m_R_*(*q*_1_,2,0) of interpolation point *m_F_*(*q*_1_,2), *m_F_*(*q*_1_,1) for existing *q*_1_~*q*_2_ is collision-free; (**b**) the path between midpoint *m_R_*(*q*_0_,2*,*1) of *m_F_*(*q*_0_,1), *m_R_*(*q*_0_,2,0) and midpoint *m_R_*(*q*_1_,2,1) of *m_R_*(*q*_1_,2,0), *m_F_*(*q*_1_,1) is not collision-free; (**c**) collision-free interpolation point *m_R_*(*q*_0_,2,0), *m_R_*(*q*_1_,2,0) is closest to the obstacle (when *d* < *ε*); (**d**) rewiring: interpolation points *m_R_*(*q*_0_,2,0) and *m_R_*(*q*_1_,2,0) are inserted between the existing paths *q*_0_~*q*_2_.

As shown in Figure 13, a collision-free interpolation point is found while descending in the direction of the midpoint (*q*_1_) among the three points (a random interpolation point (*q*_0_), the next interpolation point (*q*_1_) of *q*_0_ and the next interpolation point (*q*_2_) of *q*_1_). From the interpolation point, it ascends again in the direction of the obstacle as far as possible (*d* >= *ε*), and a waypoint collision-free from obstacle is found between the interpolation point and the waypoint (*m_F_*(*q*_0_)~*q*_1_, *q*_1_~*m_F_*(*q*_1_)).

Accordingly, the proposed method can obtain a path that is close to optimal compared to the existing PTPMI [17] method.

The interpolation point *m_r_* follows Equation (4):

(4) $$m_{R}\left(q_{i},k,u\right)=\left\{\begin{array}{c} \left(\frac{m_{R}\left(q_{i},k,u-1\right)\cdot x+m_{F}\left(q_{i},k-1\right)\cdot x}{2},\frac{m_{R}\left(q_{i},k,u-1\right)\cdot y+m_{F}\left(q_{i},k-1\right)\cdot y}{2}\right),\;\;u>0\\ \;\;\;\;\;\;\;\left(\frac{m_{F}\left(q_{i},k\right)\cdot x+m_{F}\left(q_{i},k-1\right)\cdot x}{2},\frac{m_{F}\left(q_{i},k\right)\cdot y+m_{F}\left(q_{i},k-1\right)\cdot y}{2}\right),\;\;u=0 \end{array}\right.\;\;\left(u\in N\right)$$

The *u*-th interpolated point in the direction of the obstacle from the *k*-th interpolation point *m_F_*(*q_i_*,*k*) of a random waypoint *q_i_* is called *m_R_*(*q_i_*, *k*, *u*) (the value of *k* is fixed). At this time, if *u* is 0, it becomes the midpoint of *m_F_*(*q_i_*, *k*) and *m_F_*(*q_i_*, *k* − 1). If *u* is 1, it is the midpoint of *m_R_*(*q_i_*, *k*, 0) and *m_F_*(*q_i_*, *k* − 1). That is, *m_R_*(*q_i_*, *k*, *u*) (*u* > 0) becomes the midpoint between *m_R_*(*q_i_*, *k*, *u* − 1) and *m_F_*(*qi*, *k* − 1). For reference, *d* becomes (*d_k+u_*_−1_)/2. Here, (when *u* is 0) *m_F_*(*q_i_*, *k*) (and *m_F_*(*ξ*^2^(*q_i_*),*k*)) goes down in the *ξ*(*q_i_*) direction and becomes the first obstacle collision point. *m_F_*(*q_i_*, *k* − 1) (and *m_F_*(*ξ^2^*(*q_i_*), *k* − 1)) is the point at which it did not collide with the last obstacle. Therefore, Equation (4) interpolates within the region between the obstacle collision point and the obstacle non-impact collision point.

*m_R_*(*q_i_*, *k*, *u*) in Equation (4) can also be expressed as Equation (7) through Equations (5) and (6):

(5) $$\frac{m_{F}\left(q_{i},k\right)+m_{F}\left(q_{i},k-1\right)}{2}=\frac{3\left(m_{F}\left(q_{i},k\right)\right)-\zeta \left(q_{i}\right)}{2}$$

(6) $$\frac{m_{R}\left(q_{i},k,u-1\right)+m_{F}\left(q_{i},k-1\right)}{2}=\frac{3\left(m_{R}\left(q_{i},k,u-1\right)\right)-m_{R}\left(q_{i},k,u-2\right)}{2}$$

It starts with *m_F_*(*q_i_*,*k*) and *ξ*(*q_i_*) when *u* = 0. Then, *m_R_*(*q_i_*,*k*,*u*) is found as a point that divides the line segment connecting the previous two points *m_R_*(*q_i_*,*k*,*u* − 1) and *m_R_*(*qi*,*k*,*u* − 2) in a 3:1 ratio (*k* value is fixed).

(7) $$∴m_{R}\left(q_{i},k,u\right)=\left\{\begin{array}{c} \left(\frac{3\left(m_{R}\left(q_{i},k,u-1\right)\cdot x\right)-m_{R}\left(q_{i},k,u-2\right)\cdot x}{2},\frac{3\left(m_{R}\left(q_{i},k,u-1\right)\cdot y\right)-m_{R}\left(q_{i},k,u-2\right)\cdot y}{2}\right),\;\;u>0\\ \;\;\;\;\;\;\;\;\;\;\;\;\;\;\;\;\;\;\;\;\;\;\;\;\;\;\;\;\left(\frac{3\left(m_{F}\left(q_{i},k\right)\cdot x\right)-\zeta \left(q_{i}\right)\cdot x}{2},\frac{3\left(m_{F}\left(q_{i},k\right)\cdot y\right)-\zeta \left(q_{i}\right)\cdot y}{2}\right),\;\;u=0 \end{array}\right.\;$$

In the end, Equation (7) shows the same result as Equation (4), and it is more efficient in terms of the space complexity of the algorithm.

#### Pseudocode Backward Interpolation Process

Algorithm 4 presents the pseudocode of the *interpolation*() function of backward interpolation process.
**Algorithm 4** Pseudocode of Backward Interpolation Process.**Input:** *R* ← path *R* from *ForwardInterpolationProcess* *C* ← position set *C* from *ForwardInterpolationProcess* *ε* ← threshold value *ε* from *ForwardInterpolationProcess* *t* ← point index *t* from *ForwardInterpolationProcess* *f_modify_* ← boolean *f_modify_* from *ForwardInterpolationProcess* **Output:** *R* ← modified path *R* *t* ← updated point index *t*   /**/return by reference** *f_modify_* ← result of boolean *f_modify_*   /**/return by reference** **Initialize:** *q_child_* ← *t*-th point in *R* *q_parent_* ← next point of *q_child_* in *R* *q_ancestor_* ← next point of *q_parent_* in *R***Procedure***interpolation***from***ForwardInterpolationProcess***Begin**1*d* ← altitude of triangle consisting of *q_child_*, *q_parent_*, and *q_ancestor_* with base<*q_child_*, *q_ancestor_*>2*m_a_* ← midpoint between *q_child_* and *q_parent_*3*m_b_* ← midpoint between *q_parent_* and *q_ancestor_*4**While*****true*****Do**5    **If** *d* **>=** *ε* **Then**6       **If not** *isTrapped*(*m_a_*, *m_b_*, *C*) **Then**7          *R* ← **Delete** path<*q_child_*, *q_parent_*> and path<*q_parent_*, *q_ancestor_*> from *R*
8          *m_backA_* ← external division point of line segment<*q_parent_*, *m_a_*> with the ratio 3:19          *m_backB_* ← external division point of line segment<*q_parent_*, *m_b_*> with the ratio 3:110          **While *true* Do**11              *m_freeA_* ← *m_a_*12              *m_freeB_* ← *m_b_*13              **If not** *isTrapped*(*m_backA_*, *m_backB_*, *C*) **Then**14                 *m_a_* ← *m_backA_*15                 *m_b_* ← *m_backB_*16              **Else**
*  **Break***17              *d* ← *d*/218              **If not** *d* **>=** *ε* **Then *Break***19              *m_backA_* ← external division point of line segment <*m_freeA_*, *m_a_*> with                    the ratio 3:120              *m_backB_* ← external division point of line segment <*m_freeB_*, *m_b_*> with                    the ratio 3:121          *R* ← **Insert** path<*q_child_*, *m_a_*>, path<*m_a_*, *m_b_*> and path<*m_b_*, *q_ancestor_*> to *R*22          *f_modify_* ← ***true***23          ***Break***24       **Else**25          *d* ← *d*/226          *m_a_* ← midpoint between *m_a_* and *q_parent_*27          *m_b_* ← midpoint between *m_b_* and *q_ancestor_*28    **Else**29       *t* ← *t* + 130       ***Break*****End**

The input value of the *interpolation*() function of backward interpolation process consists of path *R*, obstacle area information *C*, waypoint index *t* and path modification *f_modify_*.

Compared to the *interpolation*() function in forward interpolation process, lines 8–20 have been inserted in this *interpolation*() function. These contents are interpolated again in the direction of the obstacle after the unidirectional (*q_parent_* direction) interpolation is completed (when collision-free from the obstacle). From the 8th line, *m_backA_* is the point where the line segment connecting *q_parent_* and *m_a_* is externalized in a 3:1 ratio, *m_backB_* is the point where the line connecting *q_parent_* and *m_b_* is externalized in a 3:1 ratio, *m_freeA_* is *m_a_* and *m_freeB_* is *m_b_*. If the route between *m_backA_* and *m_backB_* is collision-free from the obstacle (*isTrapped*()), *m_a_* is updated to *m_backA_* and *m_b_* to *m_backB_*. If it is not collision-free from the obstacle, based on the current *m_a_* and *m_b_*, a path connecting *q_child_* and *m_a_*, a path connecting *m_a_* and *m_b_* and a path connecting *m_b_* and *q_ancestor_* are inserted, and the function is terminated. In the case of being collision-free from the obstacle, if *m_a_* and *m_b_* are updated, *d* becomes 1/2. If *d* is less than *ε*, the value of *t* is incremented by 1 and the function terminates. Otherwise, *m_backA_* is updated to the point where the line segment connecting *m_freeA_* and *m_a_* is externalized in a 3:1 ratio, *m_backB_* is updated to the point where the line segment connecting *m_freeB_* and *m_b_* is externalized in a 3:1 ratio, *m_freeA_* is updated to *m_a_* and *m_freeB_* to *m_b_*, and the previous process is repeated.

### 3.3. Overview of Bidirectional Interpolation Method

**Figure 14:**
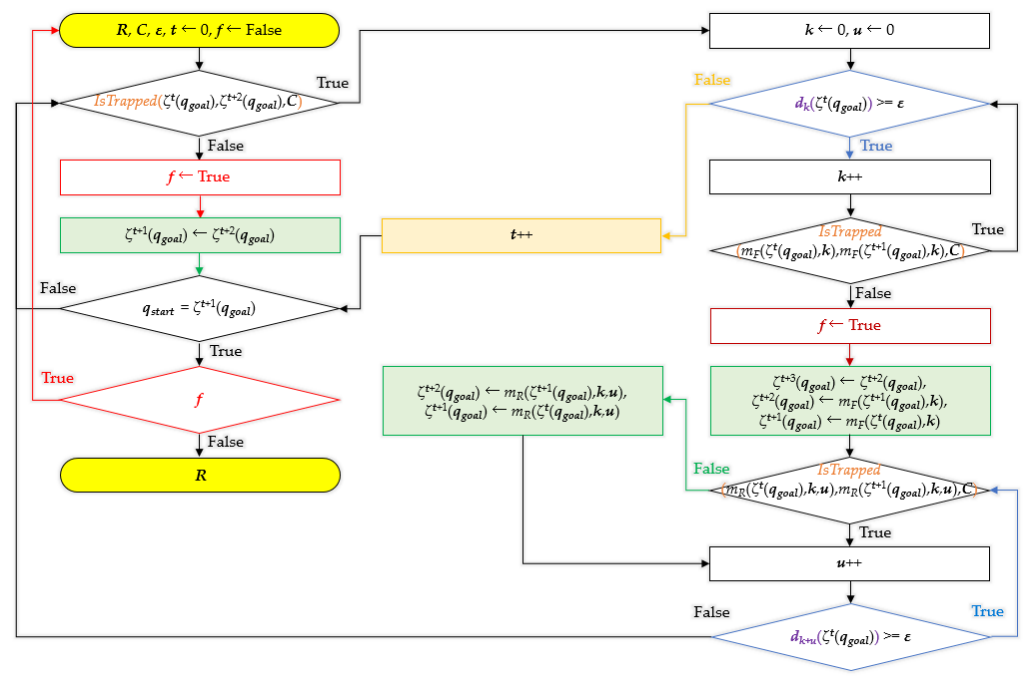
Flowchart of bidirectional interpolation method.

Figure 14 shows the overall flowchart of bidirectional interpolation method. Here, *ξ*^t^ (*q_goal_*) means the *t*-th next waypoint from the starting point *q_goal_* of the path *R*, and *ξ^t+n^*(*q_goal_*) means the *n*-th next waypoint in the *ξ^t^*(*q_goal_*). That is, there are n waypoints between *ξ^t^*(*q_goal_*) and *ξ^t+n^*(*q_goal_*).

**Figure 15:**
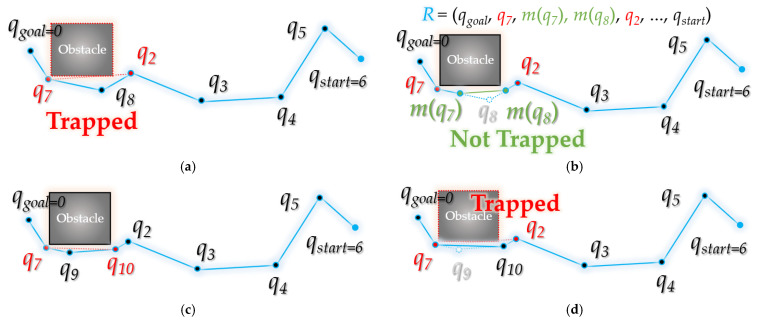

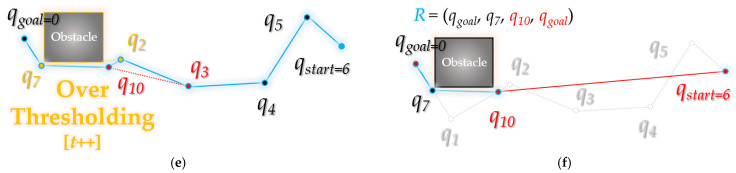
Process of bidirectional interpolation method: (**a**) (*t =* 1) between the waypoints *q*_7_ and *q*_2_ is not collision-free from the obstacle; (**b**) find the interpolation point *m*(*q*_7_) between *q*_7_ and *q*_8_ and the interpolation point *m*(*q*_8_) between *q*_8_ and *q*_2_, insert the interpolation points between the paths and delete the *q*_8_ path; (**c**) the route between *q*_7_ and *q*_10_ (from *m*(*q*_8_)) is collision-free from the obstacle, so join the path and delete path *q*_9_ (from *m*(*q*_7_)); (**d**) the route between *q*_7_ and *q*_2_ is not collision-free from the obstacle; (**e**) in the process of interpolation between *q*_7_ and *q*_2_, assuming that *d* becomes smaller than *ε*, move the focusing point (*ξ^t^*(*q_goal_*)) to the next point (*t* ← *t +* 1) and (*t* = 2). The route between *q*_10_ and *q*_3_ is collision-free from the obstacle; (**f**) as it is collision-free from the obstacle from *q*_10_ to *q_start_*, *q*_10_ and *q_start_* are connected, the waypoint between them is deleted.

Figure 15 shows, in detail, the intermediate steps in the process followed by bidirectional interpolation method to correct the planned path *R* from the starting point *q_goal_* to the destination point *q_start_*.

Figure 15a starts when the waypoint index *t* is 1. That is, as *ξ^t^*(*q_goal_*) is *ξ*(*q_goal_*), it becomes *q*_7_ in the figure. As *q*_7_ and the next waypoint *q*_2_ are not collision-free from the obstacle, interpolation proceeds. Figure 15b shows that the interpolation point *m*(*q*_7_) of *q*_7_*~q*_8_ and the interpolation point *m*(*q*_8_) of *q*_8_*~q*_2_ are free from obstacle collision. This is a case where the vertical distance *d* between the obstacle and the line segment formed by the interpolation points is smaller than the set threshold *ε*. The interpolation points *m*(*q*_7_) and *m*(*q*_8_) are inserted between the existing paths *q*_7_ to *q*_2_, and the path is modified. Existing paths *q*_7_ to *q*_8_ and *q*_8_ to *q*_2_ are deleted, and paths *q*_7_ to *m*(*q*_7_), *m*(*q*_7_) to *m*(*q*_8_), and *m*(*q*_8_) to *q*_2_ are inserted. In Figure 15c, the existing paths *q*_7_*~q*_9_*, q*_9_*~q*_10_ are deleted and *q*_7_*~q*_10_ is inserted because the distance between *q*_7_ and *q*_10_ is free from obstacle collision. In this case, *q*_9_ and *q*_10_ refer to *m*(*q*_7_) and *m*(*q*_8_) in Figure 15b. In Figure 15d, interpolation is performed for *q*_7_*~q*_10_ and *q*_10_*~q*_2_ because the line between *q*_7_ and *q*_2_ is not collision-free from the obstacle. Accordingly, index *t* becomes 2, and the focused waypoint becomes *q*_10_, which is *ξ^2^*(*q_goal_*). At this time, it can be seen that the space between *q*_10_ and *q*_3_ is free from obstacle collision. Figure 15f shows that all the waypoints on the path *q*_10_~*q_start_* are free from obstacle collision, so the existing path between *q*_10_~*q_start_* is deleted, and a path that connects *q_start_* in a straight line is inserted in *q*_10_. Finally, the path *R* is modified to (*q_goal_*, *q*_7_, *q*_10_, *q_goal_*).

## 4. Experimental Results

To check the performance of the bidirectional interpolation method proposed in this paper, the path planning results between visibility graph, RRT-connect, PTPMI and bidirectional interpolation method upon various environments were compared through simulation.

The performance measure is the average path length (px) and planning time (ms) until the first complete path is created when each algorithm (excluding visibility graph algorithm) is repeated 100 times (sampling location changes with every trial).

### 4.1. Experimental Environment

This section introduces the environment map used in the simulation and the computer specifications (i.e., hardware) for the simulation.

**Figure 16:**
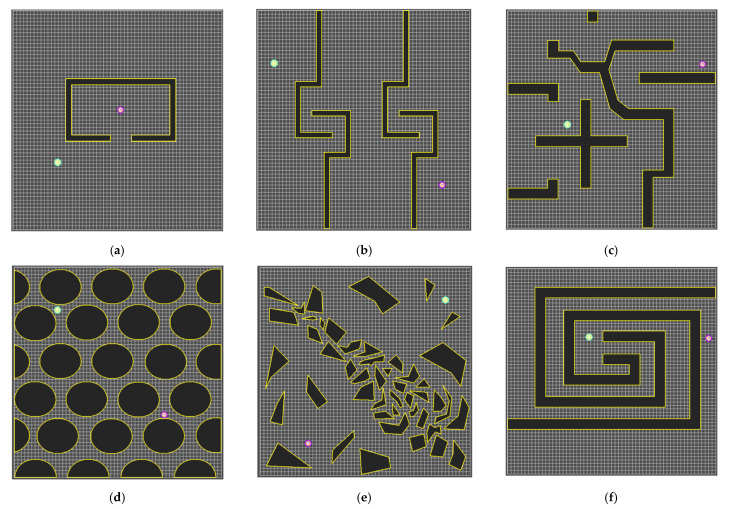
Environmental maps for the experiment: (**a**) Map 1; (**b**) Map 2; (**c**) Map 3; (**d**) Map 4; (**e**) Map 5; (**f**) Map 6.

Figure 16 shows the six environmental maps used in the experiment. Here, the green circle (S) refers to the starting point, and the purple circle (G) refers to the destination point. A black polygon with a yellow border (blue in the experimental results) indicates an obstacle. The size of all environment maps is 600 × 600 px, and the step length is 30 px.

Various environmental maps were considered and utilized to gauge the performance of bidirectional interpolation method. The environment maps used are important because the results of the performance measurement expected during the experiment are different depending on their composition, i.e., the number, arrangement and shape of the obstacles within the map. In this paper, the six environmental maps shown in Figure 16 are used to verify the performance of bidirectional interpolation method. These maps are part of the experimental environment [27] proposed by Jihee Han in 2017, and the following characteristics and efficiency of performance measures are expected for each map. Figure 16a shows Map 1, an environment in which the completeness of the path planning method can be easily verified, which is also an environment mainly used to show the local minima problem solving in the potential field algorithm [28]. Figure 16b shows Map 2, in which the optimality and completeness of the path planning method can be verified. Figure 16c shows Map 3, which is suited to verifying the optimality and completeness. Figure 16d shows Map 4, which is suited to verifying the optimality of the path planning method, as well as the planning time, because it is composed of obstacles (50 squares) that resemble a curved shape. Figure 16e shows Map 5, which is an environment in which it is easy to comprehensively verify the optimality and completeness of the path planning method as well as the planning time. Figure 16f shows Map 6, in which it is easy to verify the completeness and planning time of the path planning method. Furthermore, Map 6 is an environment that is unfavorable to sampling-based path planning methods such as the RRT algorithm.

As the sampling-based path planning method relies on probabilistic completeness, the number of sampling times and the planning time required increase considerably as there are narrow or few entrances in the direction to the destination.

Table 1 summarizes the performance of the computer used in the simulation. The simulator used for the simulation was developed based on C# WPF (Microsoft Visual Studio Community 2019 Version 16.1.6 Microsoft .NET Framework Version 4.8.03752), and only a single thread was used for calculations except for the visual part. There may be differences in planning time during simulation depending on computer performance. Therefore, in the experiment in this study, the planning time is compared not absolutely but relatively, based on the RRT-connect algorithm.

### 4.2. Experimental Results and Analysis for Each Map

In this section, the experimental results of applying the algorithms to the environment map presented in Figure 16 are stated and analyzed. The algorithms used are visibility graph, RRT-connect, triangular-RRT-connect, PTPMI and the proposed algorithm. PTPMI and the proposed algorithm were applied to the path created by RRT-connect, and the *ε* value was set to 50, 30 and 10 px, respectively. As *ε* requires a higher amount of computation as it decreases, it was set to an appropriate value nearby depending on the step length (30 px) of the experimental environment.

The experimental results will show the planned path for each algorithm for each map and show the piecewise linear shape of the path. In addition, as the results of the visibility graph for each map are presented together, the optimality of each algorithm could be visually confirmed.

The contents to be checked through the table are the path length and planning time, which are performance measures. The length of the path created through each algorithm and the relative ratio for the length of the path created by the visibility graph were considered.

PTPMI and the proposed algorithm are methods for post-processing the generated path. In this study, given that PTPMI and the proposed algorithm are applied based on RRT-connect, the planning time is compared based on the RRT-connect algorithm. The planning time is compared to basic RRT-connect and the difference between planning times of PTPMI and the proposed algorithm must be checked. The path length and planning time are presented in Table 2.

**Figure 17:**
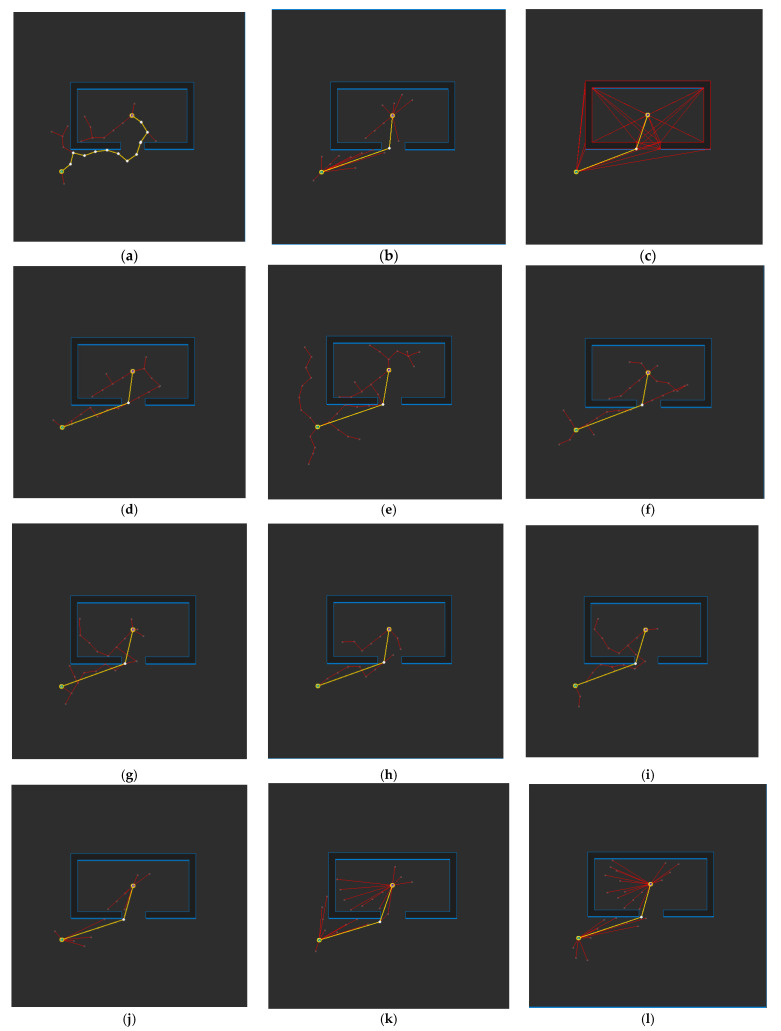

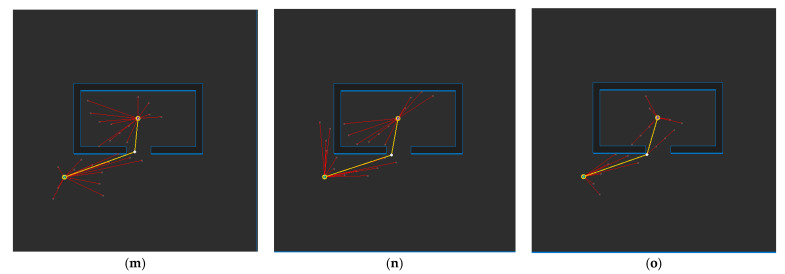
Experimental result of Map 1: (**a**) RRT-connect; (**b**) triangular-RRT-connect (**c**) visibility graph; (**d**) RRT-connect PTPMI (*ε*: 50 px); (**e**) RRT-connect PTPMI (*ε*: 30 px); (**f**) RRT-connect PTPMI (*ε*: 10 px); (**g**) RRT-connect bidirectional interpolation method (*ε*: 50 px); (**h**) RRT-connect bidirectional interpolation method (*ε*: 30 px); (**i**) RRT-connect bidirectional interpolation method (*ε*: 10 px); (**j**) triangular-RRT-connect PTPMI (*ε*: 50 px); (**k**) triangular-RRT-connect PTPMI (*ε*: 30 px); (**l**) triangular-RRT-connect PTPMI (*ε*: 10 px); (**m**) triangular-RRT-connect bidirectional interpolation method (*ε*: 50 px); (**n**) triangular-RRT-connect bidirectional interpolation method (*ε*: 30 px); (**o**) triangular-RRT-connect bidirectional interpolation method (*ε*: 10 px).

Figure 17 shows the path planning results for Map 1 for each algorithm. Looking at the generated path (yellow line), compared to Figure 17a, which is the result of RRT-connect, when PTPMI (Figure 17d–f) and the proposed algorithm (Figure 17g–i) were each applied for post-processing, the piecewise linear shape with sharp curves was reduced. In addition, it can be seen that the smaller the *ε* value, the higher the similarity with the path generated by the visibility graph (Figure 15c).

Table 2 summarizes the experimental results numerically for Map 1 based on RRT-connect among the presented environmental maps. It can be seen that the path length is the shortest when the *ε* value of the proposed algorithm is 10 (px) and is also closest to the path generated by the visibility graph (relative ratio is 257 (px)/253 (px), which is about 101%). The planning time was approximately 1 ms when the *ε* value of the proposed algorithm was 50 (px), and it takes less than 1 ms in most cases except for this case, similar to the standard RRT-connect. Thus, in Map 1, it can be confirmed that the proposed algorithm is more efficient and optimal than PTPMI.

Table 3 summarizes the experimental results numerically for Map 1 based on triangular-RRT-connect. It can be seen that the path length is the shortest when the *ε* value of the proposed algorithm is 10 (px) and is also closest to the path generated by the visibility graph (relative ratio is 257 (px)/253 (px), which is about 101%). The planning time takes less than 1 ms in all cases, similar to the standard RRT-connect. Thus, in Map 1, it can be confirmed that the proposed algorithm is a little more efficient and optimal than PTPMI.

**Figure 18:**
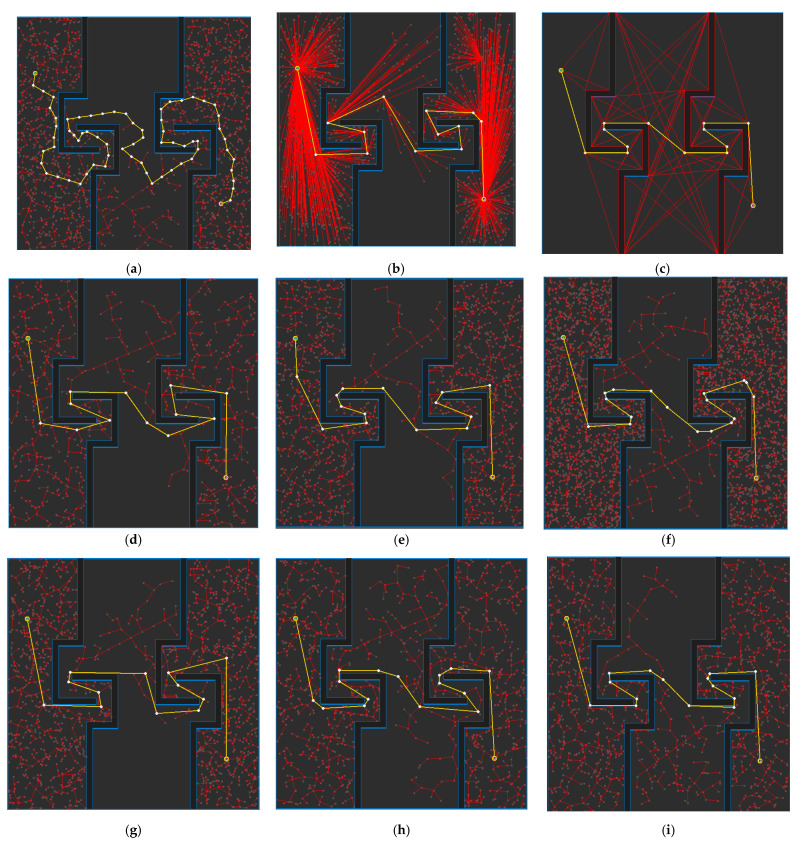

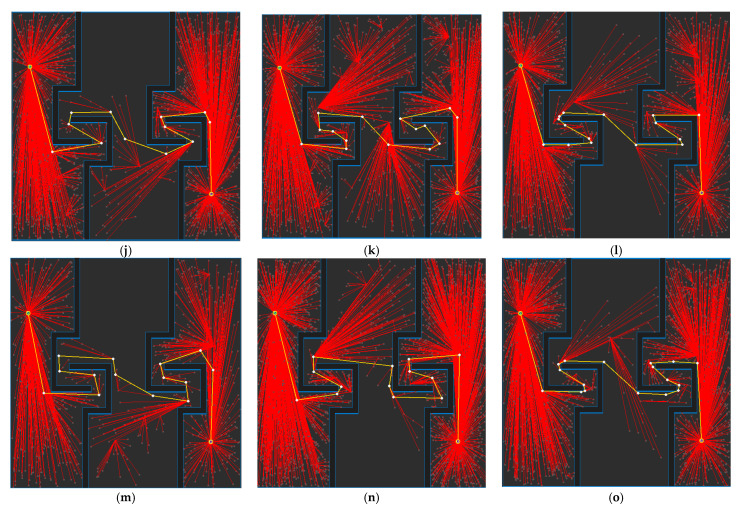
Experimental result of Map 2: (**a**) RRT-connect; (**b**) triangular-RRT-connect (**c**) visibility graph; (**d**) RRT-connect PTPMI (*ε*: 50 px); (**e**) RRT-connect PTPMI (*ε*: 30 px); (**f**) RRT-connect PTPMI (*ε*: 10 px); (**g**) RRT-connect bidirectional interpolation method (*ε*: 50 px); (**h**) RRT-connect bidirectional interpolation method *(ε*: 30 px); (**i**) RRT-connect bidirectional interpolation method (*ε*: 10 px); (**j**) triangular-RRT-connect PTPMI (*ε*: 50 px); (**k**) triangular-RRT-connect PTPMI (*ε*: 30 px); (**l**) triangular-RRT-connect PTPMI (*ε*: 10 px); (**m**) triangular-RRT-connect bidirectional interpolation method (*ε*: 50 px); (**n**) triangular-RRT-connect bidirectional interpolation method (*ε*: 30 px); (**o**) triangular-RRT-connect bidirectional interpolation method (*ε*: 10 px).

Figure 18 shows the path planning results for Map 2 for each algorithm. Looking at the generated path (yellow line), compared to Figure 18a, which is the result of RRT-connect, when PTPMI (Figure 18d–f) and the proposed algorithm (Figure 18g–i) were each applied for post-processing, the piecewise linear shape with sharp curves was reduced. In addition, it can be seen that the smaller the *ε* value, the higher the similarity with the path generated by the visibility graph (Figure 16c).

Table 4 summarizes the experimental results numerically for Map 2 based on RRT-connect. The path length is the shortest when the *ε* value of the proposed algorithm is 10 (px) and is closest to the visibility graph (relative ratio is 1223/1172, which is about 104%). It can be seen that the planning time takes longer than RRT-connect when post-processing techniques are applied. However, as expected, as the value of *ε* decreased, PTPMI and the proposed algorithm did not take longer. Rather, it can be seen that in the proposed algorithm, which requires more steps than PTPMI, the case where *ε*: 10 (px) (which is expected to take the longest time) shows the smallest difference from RRT-connect. This deviation in planning time is not due to any issues related to the post-processing technique, but is due to the random sampling effect of the RRT-like algorithms. In other words, it is difficult to find a solution for Map 2 using the RRT-based algorithm. In summary, in Map 2, it was confirmed that the proposed algorithm guarantees the optimality of the path length compared to other algorithms.

Table 5 summarizes the experimental results numerically for Map 2 based on triangular-RRT-connect. The path length is the shortest when the *ε* value of the proposed algorithm is 10 (px) and is closest to the visibility graph (relative ratio is 1229/1172, which is about 105%). However, as expected, as the value of *ε* decreased, PTPMI and the proposed algorithm did not take longer. Even some results (*ε*: 30 px, 10 px) have a shorter planning time than triangular-RRT-connect. Rather, it can be seen that in the proposed algorithm, which requires more steps than PTPMI, the case where *ε*: 10 (px) (which is expected to take the longest time) shows shorter time than triangular-RRT-connect. This difference in planning time is not due to any issues related to the post-processing technique, but is due to the random sampling effects of the RRT-like algorithms. In other words, it is more difficult to find a solution for Map 2 using the RRT-based algorithm. In summary, in Map 2, it was confirmed that the proposed algorithm guarantees the optimality of the path length compared with other algorithms.

**Figure 19:**
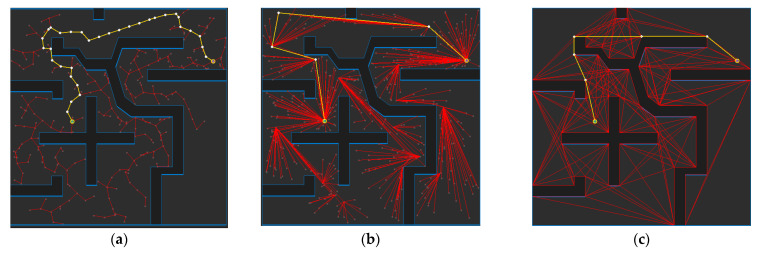

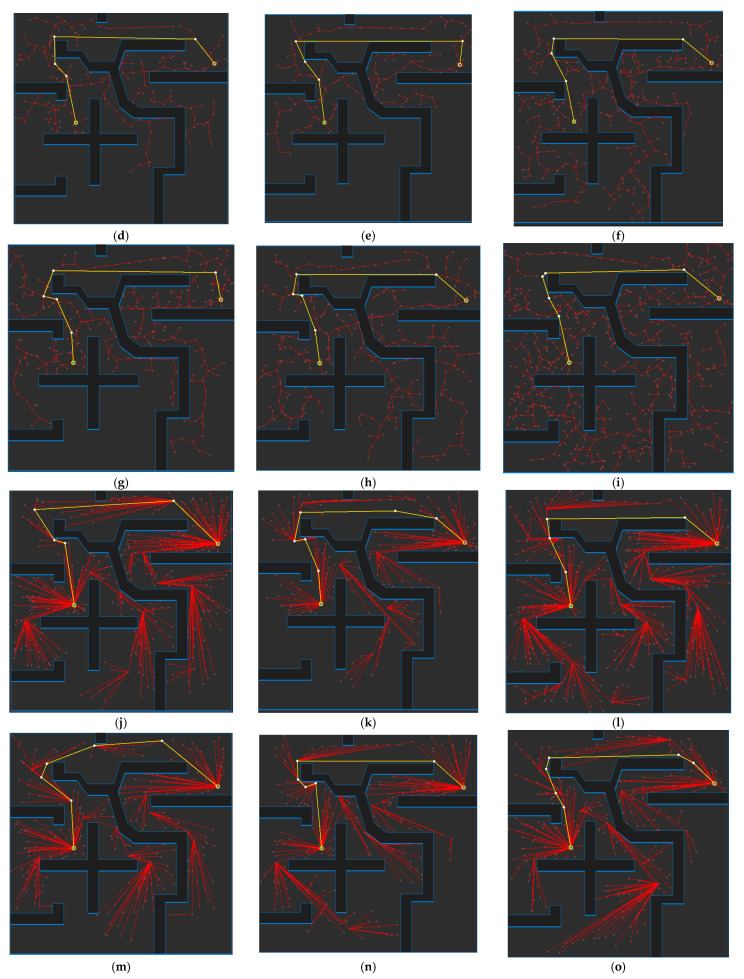
Experimental result of Map 3: (**a**) RRT-connect; (**b**) triangular-RRT-connect; (**c**) visibility graph; (**d**) RRT-connect PTPMI (*ε*: 50 px); (**e**) RRT-connect PTPMI (*ε*: 30 px); (**f**) RRT-connect PTPMI (*ε*: 10 px); (**g**) RRT-connect bidirectional interpolation method (*ε*: 50 px); (**h**) RRT-connect bidirectional interpolation method (*ε*: 30 px); (**i**) RRT-connect bidirectional interpolation method (*ε*: 10 px); (**j**) triangular-RRT-connect PTPMI (*ε*: 50 px); (**k**) triangular-RRT-connect PTPMI (*ε*: 30 px); (**l**) triangular-RRT-connect PTPMI (*ε*: 10 px); (**m**) triangular-RRT-connect bidirectional interpolation method (*ε*: 50 px); (**n**) triangular-RRT-connect bidirectional interpolation method (*ε*: 30 px); (**o**) triangular-RRT-connect bidirectional interpolation method (*ε*: 10 px).

Figure 19 shows the path planning results for Map 3 for each algorithm. Looking at the generated path (yellow line), compared to Figure 19a, which is the result of RRT-connect, when PTPMI (Figure 19d–f) and the proposed algorithm (Figure 19g–i) were each applied for post-processing, the piecewise linear shape with sharp curves was reduced. In addition, it can be seen that the smaller the *ε* value, the higher the similarity with the path generated by the visibility graph (Figure 17c).

Table 6 summarizes the experimental results numerically for Map 3 based on RRT-connect. The case where the *ε* value of the proposed algorithm is 10 (px) results in the shortest path length compared to other cases and is closest to the path generated by the visibility graph (the relative ratio is 726/714, which is about 102%). It can be seen that the planning time of RRT-connect is shorter than that of PTPMI and BPTPMI. The biggest difference from RRT-Connect occurs when using the proposed algorithm with *ε*: 10 px. However, the difference between the values is very insignificant at 3 ms. Furthermore, the shortest planning time occurred when using the proposed algorithm with *ε*:50 px. At this time, compared to RRT-connect, the path was reduced by 218 px, and compared to PTPMI, it was reduced by 4 px. This means that for Map 3, the proposed algorithm guarantees optimality compared to other algorithms and has a similar planning time to basic RRT-connect.

Table 7 summarizes the experimental results numerically for Map 3 based on triangular-RRT-connect. The case where the *ε* value of the proposed algorithm is *ε*: 10 px results in the shortest path length compared to other cases and is closest to the path generated by the visibility graph (the relative ratio is 727/714, which is about 102%). It can be seen that the planning time of triangular-RRT-connect is shorter than that of PTPMI and BPTPMI. The biggest difference from triangular-RRT-connect occurs when using the proposed algorithm. However, the difference between the values is very insignificant at 2 ms. Furthermore, the shortest path length occurred when using the proposed algorithm with *ε*: 10 px. At this time, compared to triangular-RRT-connect, the path was reduced by 86 px, and compared to PTPMI, it was reduced by 7 px. This means that for Map 3, the proposed algorithm guarantees optimality compared with other algorithms and has a similar planning time to basic RRT-connect.

**Figure 20:**
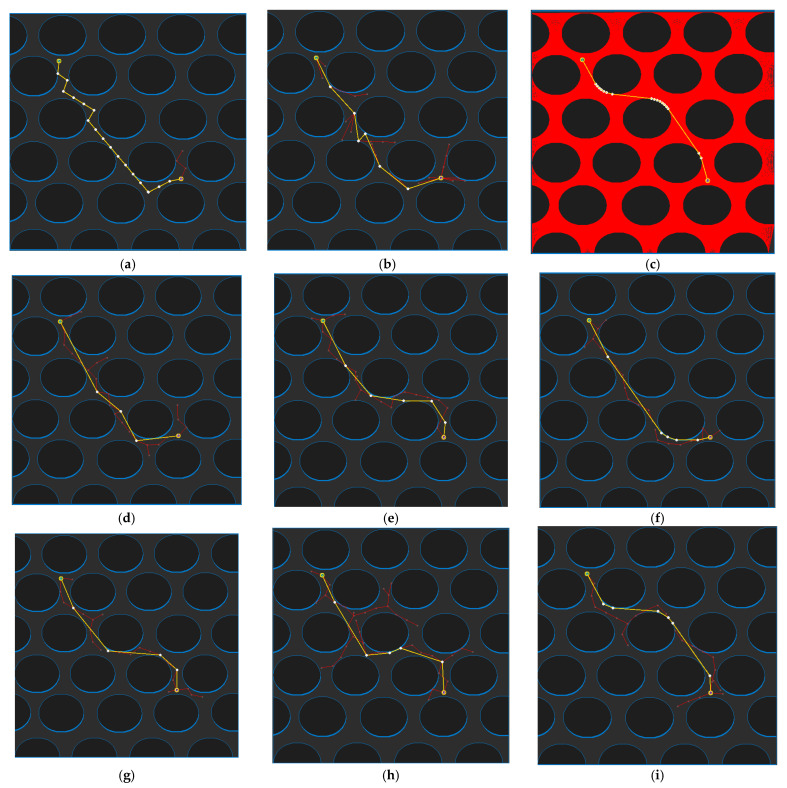

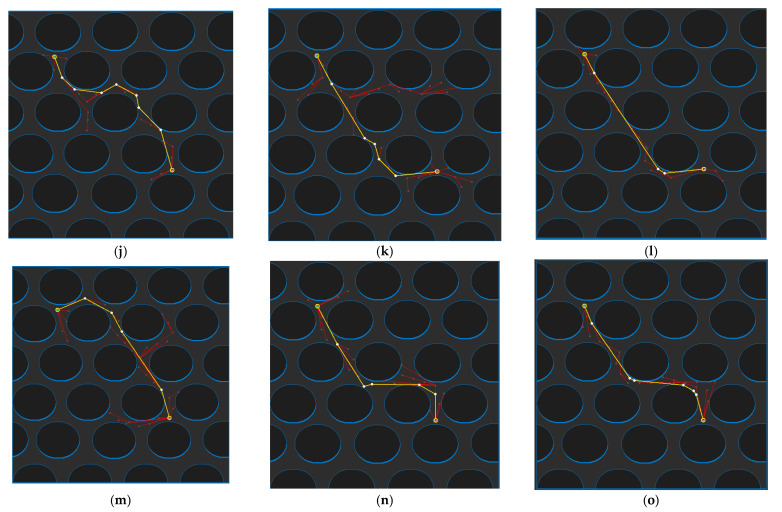
Experimental result of Map 4: (**a**) RRT-connect; (**b**) triangular-RRT-connect; (**c**) visibility graph; (**d**) RRT-connect PTPMI (*ε*: 50 px); (**e**) RRT-connect PTPMI (*ε*: 30 px); (**f**) RRT-connect PTPMI (*ε*: 10 px); (**g**) RRT-connect bidirectional interpolation method (*ε*: 50 px); (**h**) RRT-connect bidirectional interpolation method (*ε*: 30 px); (**i**) RRT-connect bidirectional interpolation method (*ε*: 10 px); (**j**) triangular-RRT-connect PTPMI (*ε*: 50 px); (**k**) triangular-RRT-connect PTPMI (*ε*: 30 px); (**l**) triangular-RRT-connect PTPMI (*ε*: 10 px); (**m**) triangular-RRT-connect bidirectional interpolation method (*ε*: 50 px); (**n**) triangular-RRT-connect bidirectional interpolation method (*ε*: 30 px); (**o**) triangular-RRT-connect bidirectional interpolation method (*ε*: 10 px).

Figure 20 shows the path planning results for Map 4 for each algorithm. Looking at the generated path (yellow line), compared to Figure 20a, which is the result of RRT-connect, when PTPMI (Figure 20d–f) and the proposed algorithm (Figure 20g–i) were each applied for post-processing, the piecewise linear shape with sharp curves was reduced. In addition, it can be seen that the smaller the *ε* value, the higher the similarity with the path generated by the visibility graph (Figure 18c).

Table 8 summarizes the experimental results numerically for Map 4 based on RRT-connect. It can be seen that the case where the *ε* value of the proposed algorithm is 10 (px) results in the shortest path length compared to other cases and is closest to the path generated by the visibility graph (relative ratio is 475/470, which is about 101%). It can be seen that the planning time of RRT-connect is shorter than that of PTPMI and bidirectional interpolation method. The biggest difference from RRT-connect occurs when using PTPMI with *ε*: 30 px. However, the difference between the values is very insignificant at 3 ms. Furthermore, the shortest planning time occurs when using the proposed algorithm with *ε*: 50 px. At this time, compared to RRT-connect, the path length is reduced by 72 px and has the same planning time as PTPMI. This means that the proposed algorithm for Map 4 guarantees optimality compared to other algorithms and has a similar planning time to basic RRT-connect.

Table 9 summarizes the experimental results numerically for Map 4 based on triangular-RRT-connect. It can be seen that the case where the *ε* value of the proposed algorithm is 10 (px) results in the shortest path length compared to other cases and is closest to the path generated by the visibility graph (relative ratio is 479/470, which is about 102%). Planning time is expressed as 1–2 ms in all cases. The biggest difference from triangular-RRT-connect occurs when using proposed algorithm with *ε*: 10 px. However, the difference between the values is very insignificant at 1 ms. This means that the proposed algorithm for Map 4 guarantees optimality compared to other algorithms and has a similar planning time to basic triangular-RRT-connect.

**Figure 21:**
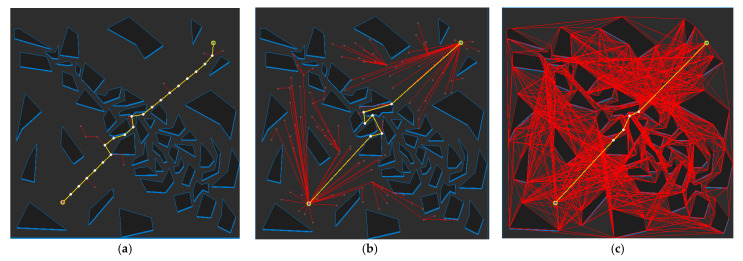

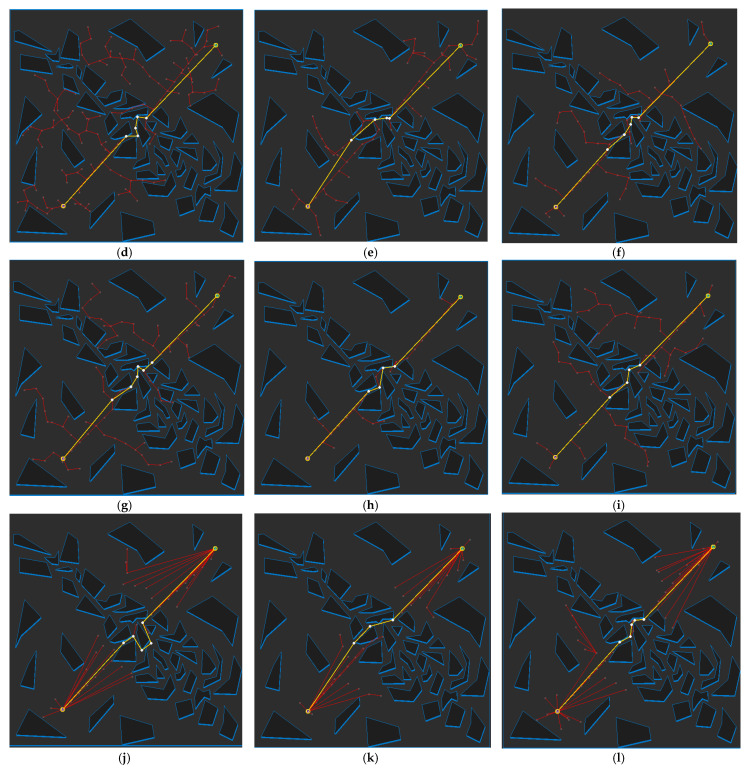

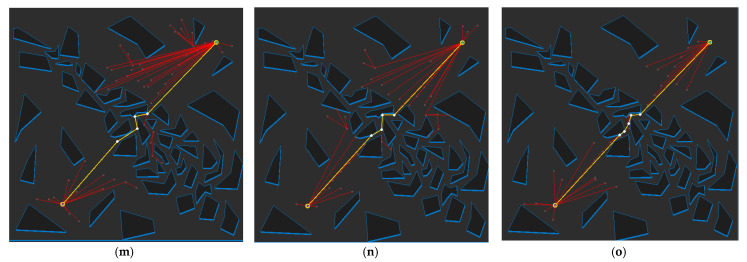
Experimental result of Map 5: (**a**) RRT-connect; (**b**) triangular-RRT-connect; (**c**) visibility graph; (**d**) RRT-connect PTPMI (*ε*: 50 px); (**e**) RRT-connect PTPMI (*ε*: 30 px); (**f**) RRT-connect PTPMI (*ε*: 10 px); (**g**) RRT-connect bidirectional interpolation method (*ε*: 50 px); (**h**) RRT-connect bidirectional interpolation method (*ε*: 30 px); (**i**) RRT-connect bidirectional interpolation method (*ε*: 10 px); (**j**) triangular-RRT-connect PTPMI (*ε*: 50 px); (**k**) triangular-RRT-connect PTPMI (*ε*: 30 px); (**l**) triangular-RRT-connect PTPMI (*ε*: 10 px); (**m**) triangular-RRT-connect bidirectional interpolation method (*ε*: 50 px); (**n**) triangular-RRT-connect bidirectional interpolation method (*ε*: 30 px); (**o**) triangular-RRT-connect bidirectional interpolation method (*ε*: 10 px).

Figure 21 shows the path planning results for Map 5 for each algorithm. Looking at the generated path (yellow line), compared to Figure 21a, which is the result of RRT-connect, when PTPMI (Figure 21d–f) and the proposed algorithm (Figure 21g–i) were each applied for post-processing, the piecewise linear shape with sharp curves was reduced. In addition, it can be seen that the smaller the *ε* value, the higher the similarity with the path generated by the visibility graph (Figure 19c).

Table 10 summarizes the experimental results numerically for Map 5 based on RRT-connect. It can be seen that the case where the *ε* value of the proposed algorithm is 10 (px) results in the shortest path length compared to other cases, and the path is closest to the one generated by the visibility graph (the relative ratio is 646/576, which is about 112%). It can be seen that the planning time of RRT-connect is shorter than that of PTPMI and bidirectional interpolation method. The biggest difference from RRT-connect occurs when using PTPMI with *ε*: 10 px. However, the difference between the values is very insignificant at 3 ms. In other situations, it can be confirmed that the planned time is always 2 ms. This means that the proposed algorithm guarantees optimality for traversing Map 5 compared to other algorithms and the planning time differs from RRT-connect by 1 ms.

Table 11 summarizes the experimental results numerically for Map 5 based on triangular-RRT-connect. It can be seen that the case where the *ε* value of the proposed algorithm is 10 (px) results in the shortest path length compared to other cases, and the path is closest to the one generated by the visibility graph (the relative ratio is 641/576, which is about 111%). Planning time is expressed as 1–2 ms in all cases. The biggest difference from triangular-RRT-connect occurs when using proposed algorithm with *ε*: 50 px. However, the difference between the values is very insignificant at 1 ms. This means that the proposed algorithm guarantees optimality compared with other algorithms and the planning time differs from triangular-RRT-connect by 1 ms.

**Figure 22:**
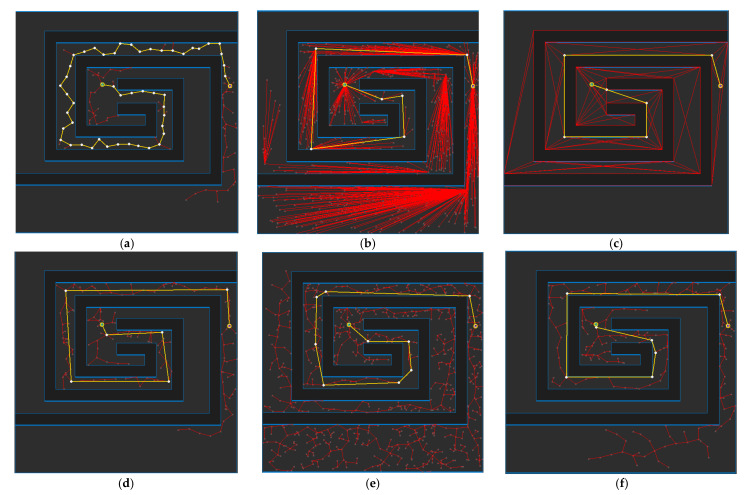

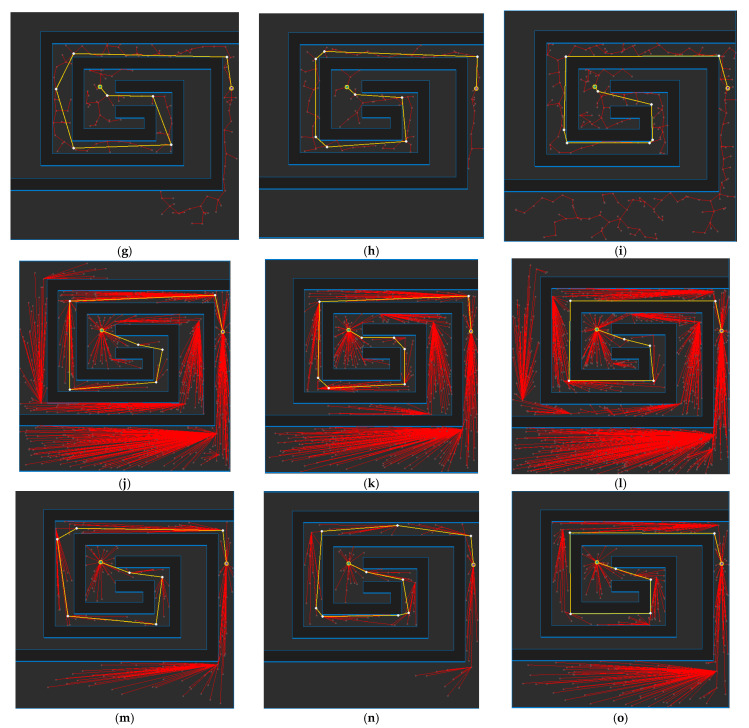
Experimental result of Map 6: (**a**) RRT-connect; (**b**) triangular-RRT-connect; (**c**) visibility graph; (**d**) RRT-connect PTPMI (*ε*: 50 px); (**e**) RRT-connect PTPMI (*ε*: 30 px); (**f**) RRT-connect PTPMI (*ε*: 10 px); (**g**) RRT-connect bidirectional interpolation method (*ε*: 50 px); (**h**) RRT-connect bidirectional interpolation method (*ε*: 30 px); (**i**) RRT-connect bidirectional interpolation method (*ε*: 10 px); (**j**) triangular-RRT-connect PTPMI (*ε*: 50 px); (**k**) triangular-RRT-connect PTPMI (*ε*: 30 px); (**l**) triangular-RRT-connect PTPMI (*ε*: 10 px); (**m**) triangular-RRT-connect bidirectional interpolation method (*ε*: 50 px); (**n**) triangular-RRT-connect bidirectional interpolation method (*ε*: 30 px); (**o**) triangular-RRT-connect bidirectional interpolation method (*ε*: 10 px).

Figure 22 shows the path planning results for Map 6 for each algorithm. Looking at the generated path (yellow line), compared to Figure 22a, which is the result of RRT-connect, when PTPMI (Figure 22d–f) and the proposed algorithm (Figure 22g–i) were each applied for post-processing, the piecewise linear shape with sharp curves was reduced. In addition, it can be seen that the smaller the *ε* value, the higher the similarity with the path generated by the visibility graph (Figure 20c).

Table 12 summarizes the experimental results numerically for Map 6 based on RRT-connect. The case where the *ε* value of the proposed algorithm is *ε*: 10 px results in the shortest path length compared to other cases and is closest to the visibility graph (the relative ratio is 1187/1165, which is about 101%). It can be seen that the planning time of RRT-Connect is shorter than that of PTPMI and the bidirectional interpolation method. The biggest difference from RRT-connect occurs when *ε*: 50 px of the algorithm is to be bounded. However, the difference between the values is very insignificant at 6 ms. Furthermore, the shortest planning time occurred when using PTPMI with *ε*: 50 px. This means that the proposed algorithm guarantees optimality compared to other algorithms, but takes an average of 4 ms longer for Map 6.

Table 13 summarizes the experimental results numerically for Map 6 based on triangular RRT-connect. The case where the *ε* value of the proposed algorithm is *ε*: 10 px results in the shortest path length compared to other cases and is closest to the visibility graph (the relative ratio is 1186/1165, which is about 102%). The planning time is similar to triangular-RRT-connect with the proposed method. The biggest difference from triangular-RRT-connect occurs when *ε*: 50 px of the algorithm is to be bounded. However, the difference between the values is very insignificant at 1 ms. Furthermore, the shortest planning time occurred when using PTPMI with *ε*: 30 px. This means that the proposed algorithm guarantees optimality compared with other algorithms.

### 4.3. Experimental Results and Analysis

In this section, the experimental results of Maps 1 to 6 are summarized.

Table 14 is a table summarizing the experimental results on the path length. It can be seen that, for all maps, the proposed algorithm creates a shorter path compared to RRT-connect. The RRT-connect algorithm generates a path whose length is approximately 138% ((150 + 157 + 140 + 122 + 132 + 127)/6) longer on average compared to the visibility graph. Similarly, PTPMI generated about 113% longer paths with *ε*: 50 px, about 108% with *ε*: 30 px and about 105% longer with *ε*: 10 px compared to the visibility graph. In the case of the proposed algorithm, it can be seen that the path generated on average is about 112% longer with *ε*: 50 px, about 108% with *ε*: 30 px and about 104% longer at *ε*: 10 px compared to the visibility graph. Thus, the proposed algorithm has a path length closer to the visibility graph as the value of epsilon decreases. Based on the *ε*: 10 px of the proposed algorithm, the average path length decreased by about 34% compared to RRT-connect, and it was improved by about 1% compared to PTPMI.

Table 15 is a table summarizing the experimental results on the path length based on triangular-RRT-connect. It can be seen that, for all maps, the proposed algorithm creates a shorter path compared to triangular-RRT-connect. The triangular-RRT-connect algorithm generates a path whose length is approximately 115% ((111 + 126 + 114 + 109 + 117 + 111)/6) longer on average compared to the visibility graph. Similarly, PTPMI generated about 112% longer paths with *ε*: 50 px, about 108% with *ε*: 30 px and about 105% longer with *ε*: 10 px compared to the visibility graph. In the case of the proposed algorithm, it can be seen that the path generated on average is about 112% longer with *ε*: 50 px, about 108% with *ε*: 30 px and about 104% longer at *ε*: 10 px compared to the visibility graph. Thus, the proposed algorithm has a path length a little closer to the visibility graph as the value of epsilon decreases. Based on the *ε*: 10 px of the proposed algorithm, the average path length decreased by about 11% compared to triangular-RRT-connect, and it was improved by about 1% compared to PTPMI.

Table 16 summarizes the experimental results on the planning time. In all maps, it can be seen that the proposed algorithm takes longer than RRT-connect. However, the difference is not large. Based on *ε*: 10 px, which was confirmed to be closest to optimality through Table 13, the biggest difference with RRT-connect, of 4 ms, occurs for Map 6.

As the proposed algorithm comprises an additional procedure to approach obstacles compared to PTPMI, it is predicted that it would require more planning time compared to PTPMI. The case in which the proposed algorithm takes the most time compared to PTPMI is when the *ε* value in Map 2 is 30 px, and the difference between the two is 8 ms. However, there are cases where the time of the proposed algorithm is reduced compared to PTPMI. In particular, for Map 2, when *ε*: 10 px, the proposed algorithm requires 223 ms, whereas PTPMI requires 250 ms, which is a reduction of 27 ms for the proposed algorithm. This may be due to the reason that the planning time of the proposed algorithm is more affected by the random sampling effects, an intrinsic problem of the RRT-series algorithm, than by the time required to process the additional procedure.

Table 17 summarizes the experimental results on the planning time. It can be seen that the proposed algorithm takes longer than triangular-RRT-connect in most maps. However, the difference is not large. Based on *ε*: 10 px, which was confirmed to be closest to optimality through Table 14, the biggest difference with RRT-Connect, of 2 ms, occurs for Map 3. Even in Maps 2 and 6, it can be seen that the time is reduced.

As the proposed algorithm comprises an additional procedure to approach obstacles compared to PTPMI, it is predicted that it would require more planning time compared to PTPMI. The case in which the proposed algorithm takes the most time compared to PTPMI is when the *ε* value in Map 2 is 50 px, and the difference between the two is 8 ms. However, there are cases where the time of the proposed algorithm is reduced compared to PTPMI. In particular, for Map 2, when *ε*: 30 px, the proposed algorithm requires 181 ms, whereas PTPMI requires 194 ms, which is a reduction of 13 ms for the proposed algorithm. Thus, it can be confirmed that the planning time of the proposed algorithm is more affected by the probabilistic integrity, an intrinsic problem of the RRT-like algorithms, than by the time required to process the additional procedure.

**Figure 23:**
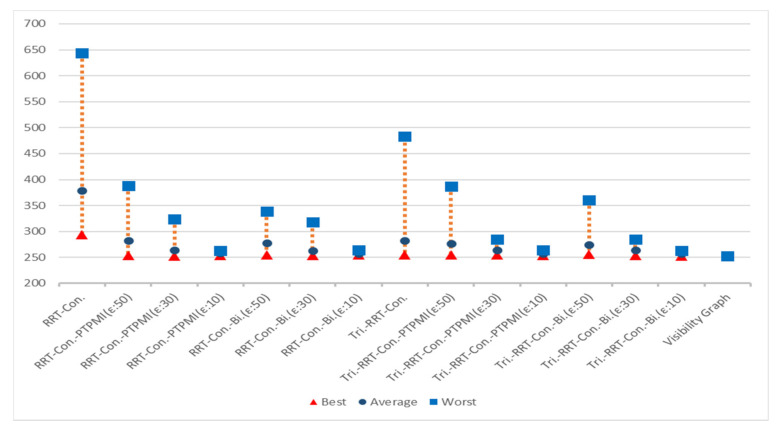
Best, average and worst path length by algorithm in Map 1.

Figure 23 shows the overall result for Map 1. The rectangle represents worst path length, the circle represents average path length and the triangle represents best path length. First of all, it can be seen that the results similar to the visibility graph appear in all cases except for RRT-connect in best path length (triangle). Average path length (circle) is shorter when the post-processing method (PTPMI or proposed method) is applied than the original RRT-connect and triangular-RRT-connect. It can be seen that worst path length (rectangle) gradually approaches average as the *ε* value decreases in the post-processing method. In other words, if the post-processing method is applied, worst path length (rectangle) is enhanced. Moreover, the proposed method enhanced worst path length better than PTPMI.

**Figure 24:**
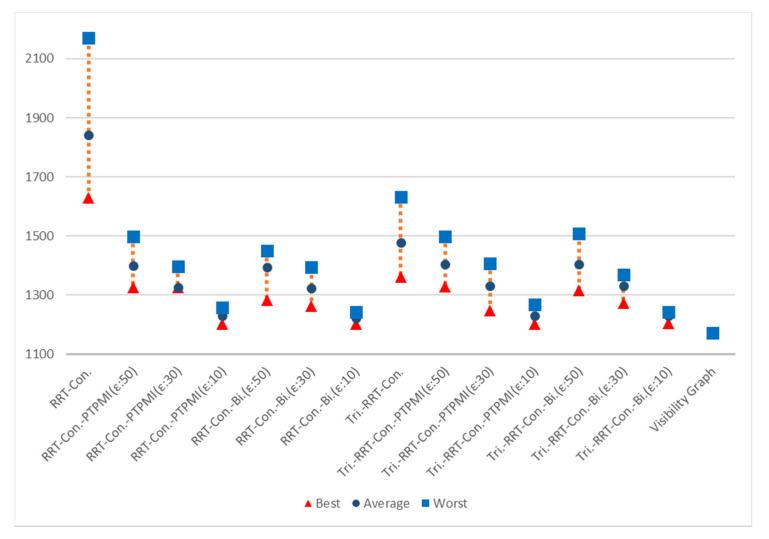
Best, average and worst path length by algorithm in Map 2.

Figure 24 shows the overall result for Map 2. It can be seen that the path length is improved in all cases (best, worst, average) when the post-processing method is applied rather than the original algorithm. In addition, as the *ε* value decreased in the post-processing method, the path length was enhanced in all cases (best, worst, average). If the *ε* value is the same, the proposed method is more enhanced than the PTPMI in all cases (best, worst, average).

**Figure 25:**
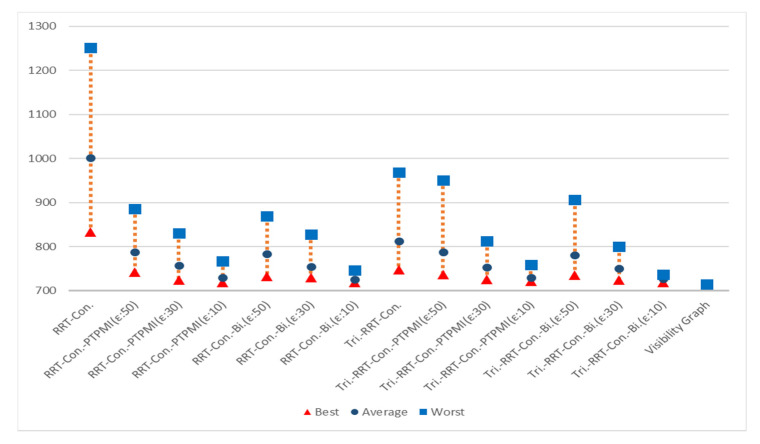
Best, average and worst path length by algorithm in Map 3.

Figure 25 shows the overall result for Map 3. It can be seen that the path length is improved in all cases (best, worst, average) when the post-processing method is applied rather than the original algorithm. In addition, as the *ε* value decreased in the post-processing method, the path length was enhanced in all cases (best, worst, average). If the *ε* value is the same, the proposed method is more enhanced than the PTPMI in all cases (best, worst, average).

**Figure 26:**
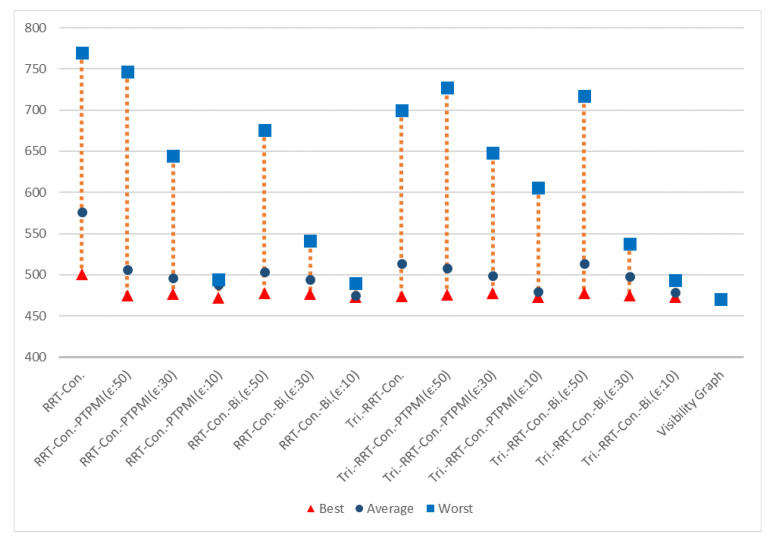
Best, average and worst path length by algorithm in Map 4.

Figure 26 shows the overall result for Map 4. It can be seen that the results similar to the visibility graph appear in all cases except for RRT-connect in best path length (triangle). When the post-processing method is applied, if *ε* value is reduced, average and worst are enhanced. Moreover, if *ε* value is the same in the post-processing method, the proposed method enhanced worst more than PTPMI.

**Figure 27:**
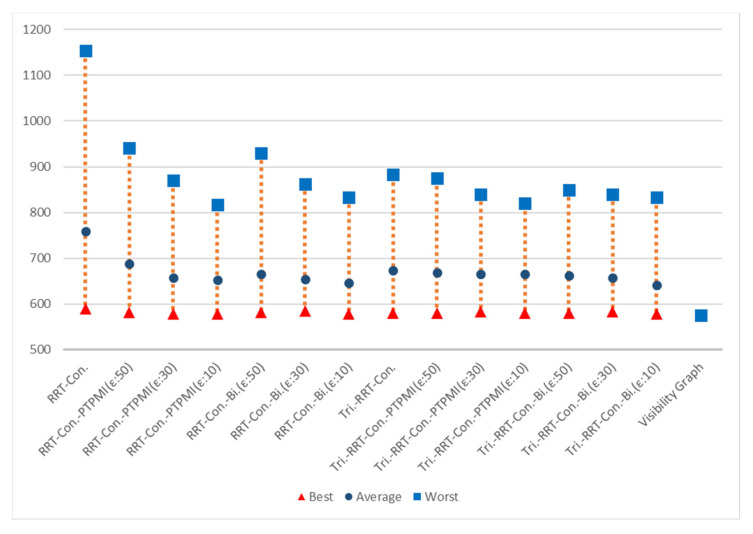
Best, average and worst path length by algorithm in Map 5.

Figure 27 shows the overall result for Map 5. It can be seen that the results similar to the visibility graph appear in all cases. When the post-processing method is applied, if *ε* value is reduced, worst is enhanced. Moreover, if *ε* value is the same in the post-processing method, the proposed method improves worst more than PTPMI.

**Figure 28:**
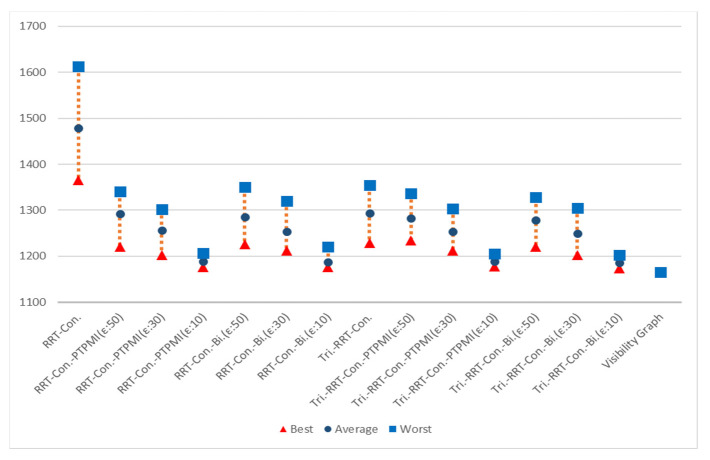
Best, Average and Worst path length by algorithm in Map 6.

Figure 28 shows the overall result for Map 6. It can be seen that the path length is improved in all cases (best, worst, average) when the post-processing method is applied rather than the original algorithm. In addition, as the *ε* value reduced in the post-processing method, the path length was enhanced in all cases (best, worst, average). If the *ε* value is the same, the proposed method is more enhanced than the PTPMI in all cases (best, worst, average).

## 5. Conclusions

In this paper, we proposed the bidirectional interpolation method. The proposed method can minimize the planning time and overcome the limit of optimality of sampling-based algorithms and kinodynamic error.

It was confirmed that bidirectional interpolation method plans a path close to the optimum when applied to the existing RRT-like algorithms through mathematical modeling. Simulations were performed to confirm the performance of bidirectional interpolation method. In six different environment maps, it was confirmed that the path length was shortened by 34% on average compared to when the basic RRT-connect algorithm was applied, and the path length was, on average, only 4% longer than the visibility graph. In addition, bidirectional interpolation method has the advantage of being applicable to all path planning methods that plan a locally piecewise linear path.

Compared with the PTPMI algorithm, it was confirmed that the proposed method shows a little better but is staggered with performance by 1–3% in terms of path length, and there was no significant difference in terms of planning time.

In most cases with all maps, the proposed method shows that worst path length was greatly reduced when the post-processing method was applied. In addition, if the *ε* value is the same, worst path length of the proposed algorithm is improved over PTPMI.

In this paper, a method applicable to the RRT-likes is proposed. However, the proposed method is a technique that can be applied to sampling-based planning algorithms such as RRT. Therefore, it can be applied in various fields where motion planning of robots is used, such as mobile robots, manipulators and drones.

## Figures and Tables

**Table 1:** Computer specifications for the simulation.

*H/W*	Spec.
CPU	Intel Core i7-6700k 4.00 GHz (8 CPUs)
RAM	32,768 MB (32 GB DDR4)

**Table 2:** Experimental result based on RRT-connect of Map 1 (the parentheses to the right of each value of path length are the relative ratios based on visibility graph (253 px)).

Performance	RRT-Connect	PTPMI Method			Bidirectional Interpolation Method		
		*ε*: 50 px	*ε*: 30 px	*ε*: 10 px	*ε*: 50 px	*ε*: 30 px	*ε*: 10 px
Path length (px)	379 (150%)	283 (112%)	264 (104%)	258 (102%)	278 (110%)	263 (104%)	257 (101%)
Planning time (ms)	<0	<0	<0	<0	1	<0	<0

**Table 3:** Experimental result based on triangular-RRT-connect of Map 1 (the parentheses to the right of each value of path length are the relative ratios based on visibility graph (253 px)).

Performance	Triangular-RRT-Connect	PTPMI Method			Bidirectional Interpolation Method		
		*ε*: 50 px	*ε*: 30 px	*ε*: 10 px	*ε*: 50 px	*ε*: 30 px	*ε*: 10 px
Path length (px)	282 (111%)	277 (109%)	264 (104%)	258 (102%)	274 (108%)	264 (104%)	257 (101%)
Planning time (ms)	<0	<0	<0	<0	<0	<0	<0

**Table 4:** Experimental result based on RRT-connect of Map 2 (the parentheses to the right of each value of path length are relative ratios based on visibility graph (1172 px)).

Performance	RRT-Connect	PTPMI Method			Bidirectional Interpolation Method		
		*ε*: 50 px	*ε*: 30 px	*ε*: 10 px	*ε*: 50 px	*ε*: 30 px	*ε*: 10 px
Path length (px)	1843 (157%)	1399 (119%)	1326 (113%)	1230 (105%)	1395 (119%)	1324 (112%)	1223 (104%)
Planning time (ms)	220	242	264	250	243	272	223

**Table 5:** Experimental result based on triangular-RRT-connect of Map 2 (the parentheses to the right of each value of path length are relative ratios based on visibility graph (1172 px)).

Performance	Triangular-RRT-Connect	PTPMI Method			Bidirectional Interpolation Method		
		*ε*: 50 px	*ε*: 30 px	*ε*: 10 px	*ε*: 50 px	*ε*: 30 px	*ε*: 10 px
Path length (px)	1478 (126%)	1405 (120%)	1331 (113%)	1230 (105%)	1404 (120%)	1331 (113%)	1229 (105%)
Planning time (ms)	195	197	194	214	205	181	194

**Table 6:** Experimental result based on RRT-connect of Map 3 (the parentheses to the right of each value of path length are relative ratios based on visibility graph (714 px)).

Performance	RRT-Connect	PTPMI Method			Bidirectional Interpolation Method		
		*ε*: 50 px	*ε*: 30 px	*ε*: 10 px	*ε*: 50 px	*ε*: 30 px	*ε*: 10 px
Path length (px)	1002 (140%)	788 (110%)	757 (106%)	729 (102%)	784 (110%)	755 (106%)	726 (102%)
Planning time (ms)	9	10	10	9	8	11	12

**Table 7:** Experimental result based on triangular-RRT-connect of Map 3 (the parentheses to the right of each value of path length are relative ratios based on visibility graph (714 px)).

Performance	Triangular-RRT-Connect	PTPMI Method			Bidirectional Interpolation Method		
		*ε*: 50 px	*ε*: 30 px	*ε*: 10 px	*ε*: 50 px	*ε*: 30 px	*ε*: 10 px
Path length (px)	813 (114%)	787 (110%)	753 (105%)	729 (102%)	781 (109%)	750 (105%)	727 (102%)
Planning time (ms)	7	10	9	10	9	9	9

**Table 8:** Experimental result based on RRT-connect of Map 4 (the parentheses to the right of each value of path length are relative ratios based on visibility graph (470 px)).

Performance	RRT-Connect	PTPMI Method			Bidirectional Interpolation Method		
		*ε*: 50 px	*ε*: 30 px	*ε*: 10 px	*ε*: 50 px	*ε*: 30 px	*ε*: 10 px
Path length (px)	576 (122%)	506 (108%)	496 (105%)	488 (104%)	504 (107%)	494 (105%)	475 (101%)
Planning time (ms)	1	2	4	2	1	3	2

**Table 9:** Experimental result based on triangular-RRT-connect of Map 4 (the parentheses to the right of each value of path length are relative ratios based on visibility graph (470 px)).

Performance	TriangularRRT-Connect	PTPMI Method			Bidirectional Interpolation Method		
		*ε*: 50 px	*ε*: 30 px	*ε*: 10 px	*ε*: 50 px	*ε*: 30 px	*ε*: 10 px
Path length (px)	514 (109%)	508 (108%)	499 (106%)	480 (102%)	514 (109%)	499 (106%)	479 (102%)
Planning time (ms)	1	1	1	2	1	2	2

**Table 10:** Experimental result based on RRT-connect of Map 5 (the parentheses to the right of each value of path length are relative ratios based on visibility graph (576 px)).

Performance	RRT-Connect	PTPMI Method			Bidirectional Interpolation Method		
		*ε*: 50 px	*ε*: 30 px	*ε*: 10 px	*ε*: 50 px	*ε*: 30 px	*ε*: 10 px
Path length (px)	759 (132%)	689 (120%)	657 (114%)	653 (113%)	666 (117%)	655 (114%)	646 (112%)
Planning time (ms)	1	2	2	4	2	2	2

**Table 11:** Experimental result based on triangular-RRT-connect of Map 5 (the parentheses to the right of each value of path length are relative ratios based on visibility graph (576 px)).

Performance	Triangular-RRT-Connect	PTPMI Method			Bidirectional Interpolation Method		
		*ε*: 50 px	*ε*: 30 px	*ε*: 10 px	*ε*: 50 px	*ε*: 30 px	*ε*: 10 px
Path length (px)	673 (117%)	669 (116%)	666 (116%)	665 (115%)	663 (115%)	658 (114%)	641 (111%)
Planning time (ms)	1	1	1	2	2	1	1

**Table 12:** Experimental result based on RRT-connect of Map 6 (the parentheses to the right of each value of path length are relative ratios based on visibility graph (1165 px)).

Performance	RRT-Connect	PTPMI Method			Bidirectional Interpolation Method		
		*ε*: 50 px	*ε*: 30 px	*ε*: 10 px	*ε*: 50 px	*ε*: 30 px	*ε*: 10 px
Path length (px)	1478 (127%)	1292 (111%)	1257 (108%)	1189 (102%)	1286 (110%)	1254 (108%)	1187 (102%)
Planning time (ms)	24	24	27	26	30	26	28

**Table 13:** Experimental result based on triangular-RRT-connect of Map 6 (the parentheses to the right of each value of path length are relative ratios based on visibility graph (1165 px)).

Performance	RRT-Connect	PTPMI Method			Bidirectional Interpolation Method		
		*ε*: 50 px	*ε*: 30 px	*ε*: 10 px	*ε*: 50 px	*ε*: 30 px	*ε*: 10 px
Path length (px)	1293 (111%)	1282 (110%)	1253 (108%)	1189 (102%)	1279 (110%)	1250 (107%)	1186 (102%)
Planning time (ms)	23	24	23	22	23	22	23

**Table 14:** Total experimental result for path length based on RRT-connect (the parentheses to the right of each value of path length are relative ratios based on the visibility graph) (unit: px).

Map No.	RRT-Connect	PTPMI Method			Bidirectional Interpolation Method			Visibility Graph
		*ε*: 50 px	*ε*: 30 px	*ε*: 10 px	*ε*: 50 px	*ε*: 30 px	*ε*: 10 px	
Map 1	379 (150%)	283 (112%)	264 (104%)	258 (102%)	278 (110%)	263 (104%)	257 (101%)	253
Map 2	1843 (157%)	1399 (119%)	1326 (113%)	1230 (105%)	1395 (119%)	1324 (112%)	1223 (104%)	1172
Map 3	1002 (140%)	788 (110%)	757 (106%)	729 (102%)	784 (110%)	755 (106%)	726 (102%)	714
Map 4	576 (122%)	506 (108%)	496 (105%)	488 (104%)	504 (107%)	494 (105%)	475 (101%)	470
Map 5	759 (132%)	689 (120%)	657 (114%)	653 (113%)	666 (117%)	655 (114%)	646 (112%)	576
Map 6	1478 (127%)	1292 (111%)	1257 (108%)	1189 (102%)	1286 (110%)	1254 (108%)	1187 (102%)	1165

**Table 15:** Total experimental result for path length based on triangular-RRT-connect (the parentheses to the right of each value of path length are relative ratios based on the visibility graph) (unit: px).

Map No.	Triangular-RRT-Connect	PTPMI Method			Bidirectional Interpolation Method			Visibility Graph
		*ε*: 50 px	*ε*: 30 px	*ε*: 10 px	*ε*: 50 px	*ε*: 30 px	*ε*: 10 px	
Map 1	282 (111%)	277 (109%)	264 (104%)	257 (101%)	274 (108%)	264 (104%)	257 (101%)	253
Map 2	1478 (126%)	1405 (120%)	1331 (113%)	1230 (105%)	1404 (120%)	1331 (113%)	1229 (105%)	1172
Map 3	813 (114%)	787 (110%)	753 (105%)	729 (102%)	781 (109%)	750 (105%)	727 (102%)	714
Map 4	514 (109%)	508(108%)	499(106%)	480(102%)	514(109%)	499(106%)	479(102%)	470
Map 5	673 (117%)	669 (116%)	666 (116%)	665 (115%)	663 (115%)	658 (114%)	641 (111%)	576
Map 6	1293 (111%)	1282 (110%)	1253 (108%)	1189 (102%)	1279 (110%)	1250 (107%)	1186 (102%)	1165

**Table 16:** Experimental result for planning time based on RRT-connect (unit: ms).

Map No.	RRT-Connect	PTPMI Method			Bidirectional Interpolation Method		
		*ε*: 50 px	*ε*: 30 px	*ε*: 10 px	*ε*: 50 px	*ε*: 30 px	*ε*: 10 px
Map 1	<0	<0	<0	<0	1	<0	<0
Map 2	220	242	264	250	243	272	223
Map 3	9	10	10	9	8	11	12
Map 4	1	2	4	2	1	3	2
Map 5	1	2	2	4	2	2	2
Map 6	24	24	27	26	30	26	28

**Table 17:** Experimental result for planning time based on triangular-RRT-connect (unit: ms).

Map No.	TriangularRRT-Connect	PTPMI Method			Bidirectional Interpolation Method		
		*ε*: 50 px	*ε*: 30 px	*ε*: 10 px	*ε*: 50 px	*ε*: 30 px	*ε*: 10 px
Map 1	<0	<0	<0	<0	<0	<0	<0
Map 2	195	197	194	214	205	181	194
Map 3	7	10	9	10	9	9	9
Map 4	1	1	1	2	1	2	2
Map 5	1	1	1	2	2	1	1
Map 6	27	24	20	22	19	22	23

## Data Availability

Not applicable.
